# Mechanical Power Density Predicts Prolonged Ventilation Following Double Lung Transplantation

**DOI:** 10.3389/ti.2023.11506

**Published:** 2023-09-20

**Authors:** Alessandro Ghiani, Nikolaus Kneidinger, Claus Neurohr, Sandra Frank, Ludwig Christian Hinske, Christian Schneider, Sebastian Michel, Michael Irlbeck

**Affiliations:** ^1^ Department of Pulmonology and Respiratory Medicine, Lung Center Stuttgart–Schillerhoehe Lung Clinic GmbH, Robert-Bosch-Hospital GmbH, Stuttgart, Germany; ^2^ Department of Medicine V, LMU University Hospital, LMU Munich, Munich, Germany; ^3^ Comprehensive Pneumology Center (CPC-M), German Center for Lung Research (DZL), Munich, Germany; ^4^ Department of Anesthesiology, Ludwig-Maximilians-University (LMU) of Munich, Munich, Germany; ^5^ Institute for Digital Medicine, University Hospital Augsburg, Augsburg, Germany; ^6^ Department of Thoracic Surgery, Ludwig-Maximilians-University (LMU) of Munich, Munich, Germany; ^7^ Clinic of Cardiac Surgery, Ludwig-Maximilians-University (LMU) of Munich, Munich, Germany

**Keywords:** lung transplantation, prolonged ventilation, ventilator weaning, extubation, mechanical power

## Abstract

Prolonged mechanical ventilation (PMV) after lung transplantation poses several risks, including higher tracheostomy rates and increased in-hospital mortality. Mechanical power (MP) of artificial ventilation unifies the ventilatory variables that determine gas exchange and may be related to allograft function following transplant, affecting ventilator weaning. We retrospectively analyzed consecutive double lung transplant recipients at a national transplant center, ventilated through endotracheal tubes upon ICU admission, excluding those receiving extracorporeal support. MP and derived indexes assessed up to 36 h after transplant were correlated with invasive ventilation duration using Spearman’s coefficient, and we conducted receiver operating characteristic (ROC) curve analysis to evaluate the accuracy in predicting PMV (>72 h), expressed as area under the ROC curve (AUROC). PMV occurred in 82 (35%) out of 237 cases. MP was significantly correlated with invasive ventilation duration (Spearman’s *ρ* = 0.252 [95% CI 0.129–0.369], *p* < 0.01), with power density (MP normalized to lung-thorax compliance) demonstrating the strongest correlation (*ρ* = 0.452 [0.345–0.548], *p* < 0.01) and enhancing PMV prediction (AUROC 0.78 [95% CI 0.72–0.83], *p* < 0.01) compared to MP (AUROC 0.66 [0.60–0.72], *p* < 0.01). Mechanical power density may help identify patients at risk for PMV after double lung transplantation.

## Graphical Abstract

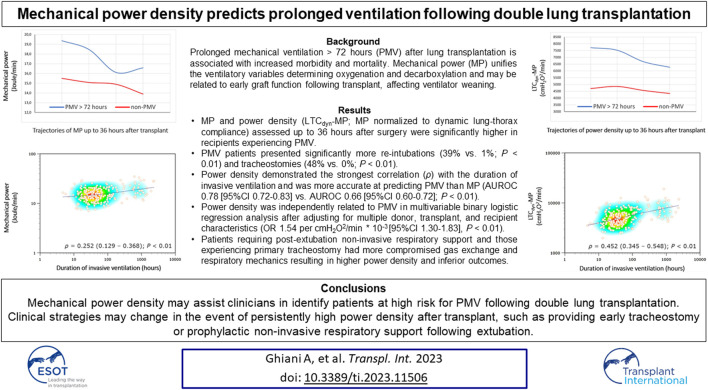

## Introduction

Lung transplantation is a life-saving treatment for patients suffering from a wide range of end-stage lung diseases. Several factors may interfere with allograft function during the early postoperative period, such as pre-existing donor and recipient-related conditions or elements of the transplant procedure causing lung injury in the donor, during the transition, or in the recipient [1]. Specifically, primary graft dysfunction (PGD), diagnosed and graded based on a chest X-ray and oxygenation levels [2], may compromise allograft function and delay ventilator weaning and time to extubation [3]. Recent studies suggest that PGD scores proposed by the international society of heart and lung transplantation (ISHLT) do not accurately predict the duration of invasive ventilation [4]. However, identifying whether a patient requires prolonged mechanical ventilation (PMV) is critical due to the risks involved, such as lower success in extubation with a subsequent higher incidence of tracheostomies, a more extended stay in the ICU, and increased in-hospital mortality [5].

Mechanical power (MP) of artificial ventilation [6], equal to the displacement work performed by the ventilator per minute, unifies the ventilatory variables that determine oxygenation (e.g., positive end-expiratory pressure) and decarboxylation (e.g., minute ventilation) and thus may be related to allograft function during the early postoperative period. Previous research indicates that MP may predict weaning outcomes in mechanically ventilated critically ill patients, with failure patients experiencing consistently longer invasive ventilation periods [7, 8]. Accordingly, we hypothesized that MP would be equally effective in predicting weaning outcomes following lung transplantation.

The study aimed to determine whether mechanical power correlates with invasive ventilation duration and predicts PMV following double lung transplantation.

## Materials and Methods

We conducted a retrospective observational study at the Ludwig-Maximilians-University (LMU) lung transplantation center (Munich, Germany). The local institutional review board approved the project (file 21-1096) per principles outlined in the Declaration of Helsinki. As the analysis was retrospective, informed consent was waived.

### Patient Selection

We screened all patients who underwent lung transplantation from January 2016 to June 2021 and focused on double lung transplant recipients mechanically ventilated through endotracheal tubes at ICU admission. Criteria for exclusion were: 1) post-operative venoarterial/venovenous extracorporeal membrane oxygenation (ECMO), 2) single-lung transplantations, 3) re-transplantations, 4) extubation delay for reasons other than weaning unreadiness and 5) re-intubation for reasons other than post-extubation respiratory failure.

### Post-operative Ventilatory Management

Lung protective mechanical ventilation is applied to all patients after transplant to prevent ventilator-induced lung injury [9–11], a factor that may contribute to PGD [12], which is particularly relevant to undersized allografts [13–15]. Protective ventilation involves tidal volumes of 6–8 mL/kg recipient-predicted body weight (PBW), plateau pressures of less than 30 cmH_2_O, and positive end-expiratory pressure (PEEP) adjusted to F_i_O_2_ without exceeding 12–14 cmH_2_O [16]. Once the level of sedation has been reduced and patients are deemed ready for ventilator weaning, they are placed on assisted ventilation, providing continuous positive airway pressure (CPAP) with low pressure support. The next step is extubation if pressure support ventilation is maintained for several hours without signs of respiratory distress. When extubated, non-invasive respiratory support may be applied in response to clinical signs of respiratory muscle fatigue and blood gas analysis showing impaired gas exchange, including nasal high-flow cannula (NHFC) and non-invasive mask ventilation (NIV) [17]. Tracheostomies are performed in cases of re-intubation or when patients cannot sustain pressure support ventilation, preventing extubation.

### Data Collection

Patients’ baseline demographics, clinical characteristics (e.g., pulmonary hemodynamics before transplant), comorbidities, procedural features in transplant (e.g., the predicted total lung capacity (pTLC) ratio [18]), and donor parameters were collected from the hospital’s electronic and Eurotransplant database. Research team members examined the radiologists’ reports for terms congruent with PGD, including pulmonary edema, reperfusion injury, and primary graft dysfunction, determining the P/F ratio based on P_a_O_2_ and FiO_2_. The 2016 ISHLT definition was used to diagnose PGD [2], focusing on PGD grades at T+72 h after transplantation among patients still receiving invasive mechanical ventilation.

We assessed the trajectories and duration of invasive ventilation after transplant (including extubations, re-intubations, and tracheostomies), and we determined the time between ICU admission and weaning readiness, defined as the first transition from pressure-controlled to pressure-support ventilation. Ventilatory variables with corresponding arterial blood gas analyses (ABGs) were recorded in each patient during pressure-controlled ventilation up to 36 h after entering the ICU or until extubation (if performed within 36 h of admission), with the median of these values used for the analysis. We also analyzed non-invasive respiratory support (NHFC and NIV) following (first) extubation, studying the number of patients treated with these techniques and the duration of treatment. Finally, we assessed the number and percentage of transplant recipients dying in the ICU.

### Ventilatory Variables and Indexes

Pressure-controlled ventilation (Evita XL, Dräger Medical GmbH, Lübeck, Germany) was used via endotracheal tubes on all patients admitted to the ICU after transplantation. Variables collected included inspired oxygen fraction (F_i_O_2_), respiratory rate, tidal volume (VT), minute ventilation, peak inspiratory airway pressure (P_peak_), and PEEP, with the following indexes calculated: P/F ratio (quotient of partial pressure of oxygen to inspired oxygen fraction), dynamic driving pressure (∆P_aw_; defined as P_peak_–PEEP), dynamic lung-thorax compliance (LTC_dyn_; defined as VT/∆P_aw_), ventilatory ratio (VR; a measure of ventilatory efficiency correlating with the pulmonary dead space fraction) [19], and MP [6] utilizing a simplified formula for pressure-controlled ventilation [20].

### Mechanical Power and Power Density

During artificial ventilation, MP is the pressure-volume work per minute provided by the ventilator, as determined by respiratory rate, tidal volume, and applied airway pressure. To account for lung dimensions interacting with MP, we normalized power to the recipients’ predicted body weight (PBW-MP) [21] and LTC_dyn_ (LTC_dyn_-MP) [7, 8]. PBW correlates with the total lung capacity of a healthy individual. Dynamic compliance with its temporal changes is a surrogate of ventilated lung volume, accounting for the force required to overcome the respiratory system’s resistance and elastance, which is critical during ventilator weaning [7, 8].

On a physical level, normalizing MP to lung volume surrogates determines the intensity of mechanical stress exerted on the respiratory system based on applied airway pressure and respiratory rate, referred to as mechanical “*power density*”. Power density, previously termed “*specific mechanical power*” [22], is equivalent to tidal “*strain energy density*” per minute, which refers to the stored energy per unit of volume resulting from a body’s linear elastic deformation [23]. Consequently, power density describes the time rate of energy transfer per unit of volume displaced and is inherently equivalent to pressure application per time. A detailed description of the calculated ventilatory indexes is provided in the online supplement (see Supplementary material).

### Classification of Outcomes

Each period of invasive mechanical ventilation (by endotracheal tube or tracheal cannula) was summarized to determine the total “*duration of invasive ventilation*” after transplant considering four clinical scenarios: 1) patients successfully extubated at the first attempt, 2) patients successfully extubated at the second or third attempt (refers to individuals experiencing extubation failure with re-intubation), 3) patients tracheotomized after one or more failed extubations, and 4) patients tracheotomized without previous extubation (referred to as primary tracheostomy) (see Supplementary Figure S1).

Although “*prolonged mechanical ventilation*” after lung transplantation has no universal definition [4, 5], the present study refers to PMV as invasive ventilation lasting longer than 72 h based on previous reports demonstrating that most patients were extubated within this period [4, 24].

“*Weaning readiness*” refers to the first transition from pressure-controlled to pressure-support ventilation, typically indicating the point where spontaneous breathing with subsequent extubation seems feasible. “*Non-invasive respiratory support*” refers to applying NHFC and/or NIV within 24 hours and for at least six hours daily following (first) extubation.

### Primary and Secondary Outcomes

The study’s primary outcome was the correlation between the duration of invasive ventilation and MP. Moreover, the ability of MP and other ventilatory indexes to predict PMV and the evaluation of patients according to the level of respiratory support required after surgery (including those with post-extubation non-invasive respiratory support and primary tracheostomy) were secondary outcomes.

### Statistical Analysis

Descriptive and frequency statistics summarized patients’ baseline characteristics. A Chi-square or Fisher’s exact test was used when comparing categorical variables. Depending on the continuous variables’ homogeneity of variance, determined by the Kolmogorov-Smirnov normality test, differences were analyzed through Student’s t-test or Mann-Whitney *U*-test. Similarly, we performed bivariate comparisons of the ventilatory variables and indexes in recipients with and without post-transplant PMV and between patients requiring varying levels of respiratory support following surgery. We conducted a (factorial) repeated-measures analysis of variance (ANOVA) to detect within-subject time effects and between-group differences in ventilatory variables’ trajectories after transplant.

We correlated the duration of invasive ventilation after lung transplant with MP and other ventilatory indexes using Spearmans’ correlation coefficient (*ρ*). The parameters’ ability to predict PMV was assessed through receiver operating characteristic (ROC) curve analysis, with diagnostic accuracy expressed as the area under the ROC curve (AUROC) [25]. In the absence of an external test cohort, we performed 2-time repeated, 5-fold cross-validation to evaluate the internal validity of these indexes [26]. We performed a multivariable binary logistic regression analysis to determine whether MP was independently associated with PMV. Recipient, transplant, and donor characteristics deemed clinically relevant *a priori* and those with a *p*-value of less than 0.2 in the univariable analysis have been utilized as input variables for multivariable model development. We performed a forward selection of variables, with Hosmer & Lemeshow test and Nagelkerke R^2^ employed to evaluate the model’s goodness-of-fit. Probabilities were expressed as odds ratios (OR) with 95% confidence intervals (95%CI).

We conducted sensitivity analyses by either redefining the threshold for prolonged ventilation (to more than 96 h and 7 days, respectively) or restricting the analysis to patients following a particular clinical scenario (as described above) [27].

Since there were no comparable studies on prolonged ventilated lung transplant recipients to determine sample size, we recruited patients to the maximum extent possible. We performed two-tailed tests; statistical significance was indicated by *p* < 0.05. The analyses were conducted with MedCalc^®^ statistical software v20.305 (MedCalc Software Ltd, Ostend, Belgium; 2023).

## Results

**FIGURE 1:**
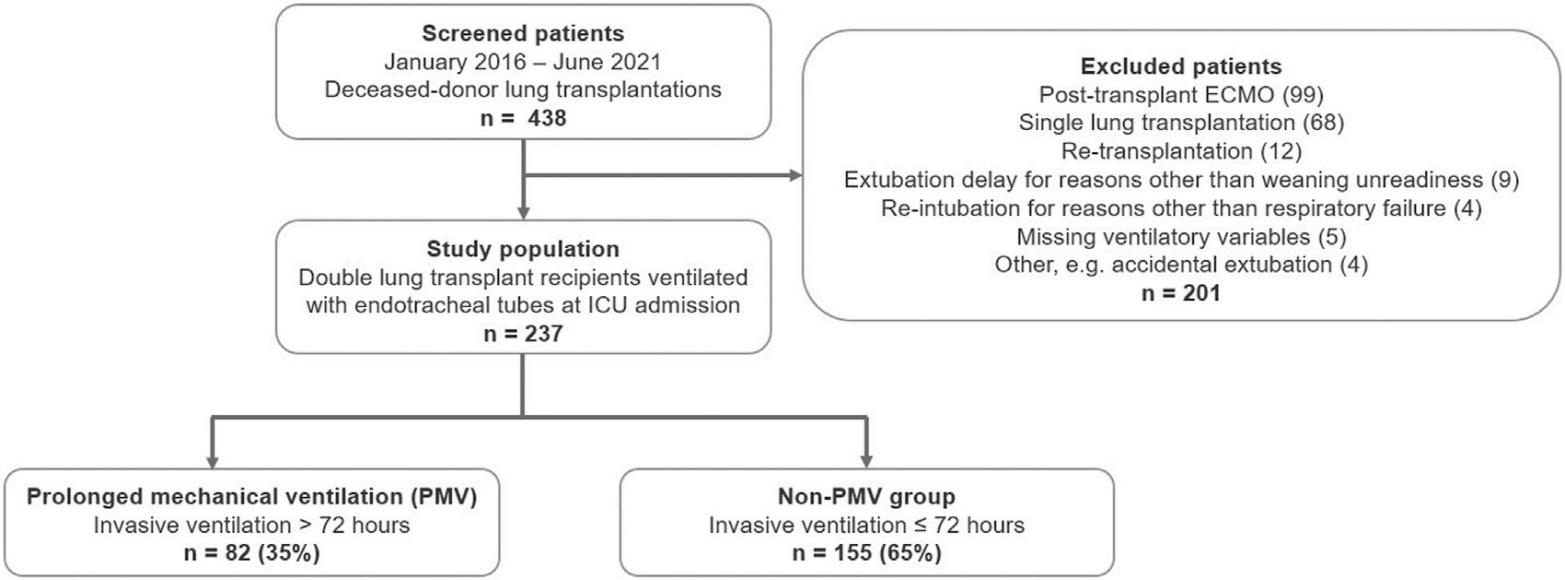
Patient flow diagram. Legend: Abbreviations. ECMO, extracorporeal membrane oxygenation; PMV, prolonged mechanical ventilation (>72 h).

Two hundred thirty-seven out of 438 screened patients (54.1%) were eligible for the study from January 2016 to June 2021. Most patients were excluded because of postoperative ECMO and single-lung transplantations in 99 and 68 cases (38.1%), respectively. Twelve patients (2.7%) had re-transplantations, 9 (2.1%) were delayed in extubation for reasons other than “weaning unreadiness” (the majority involved neurological complications, e.g., intraoperative cardiopulmonary resuscitation-induced encephalopathy), and four (0.9%) were re-intubated due to reasons other than post-extubation respiratory failure (e.g., cardiac arrest due to arrhythmia) (Figure 1).

### Baseline Characteristics

Baseline characteristics between PMV and non-PMV subjects differed regarding pulmonary hemodynamics before transplant, provision of intraoperative ECMO, and number of red cell transfusions received during transplant. PMV was more prevalent in females (58.5% versus 32.3%, *p* < 0.01), accounting for reduced recipients’ and donors’ heights and predicted body weights within the PMV group (Table 1).

**TABLE 1:** Baseline recipient, transplant, and donor characteristics.

Recipient characteristics	All patients (*n* = 237)	PMV (*n* = 82)	Non-PMV (*n* = 155)	*p*-value^a^
Age (years)	56 (47–60)	55 (47–60)	56 (47–61)	0.315^b^
Female gender	98 (41.1)	48 (58.5)	50 (32.3)	**< 0.01** ^b^
Height (m)	1.71 (1.65–1.78)	1.67 (1.60–1.75)	1.72 (1.68–1.78)	**< 0.01** ^b^
Weight (kg)	68 (55–78)	66 (52–80)	68 (58–78)	0.315^b^
Predicted body weight (kg)	66 (57–73)	60 (52–70)	68 (62–73)	**< 0.01** ^b^
Body mass index (kg/m^2^)	23.0 (19.6–26.6)	24.2 (19.5–27.4)	23.0 (19.7–25.7)	0.279^b^
Recipient pulmonary hemodynamics				
Mean pulmonary artery pressure (mmHg)	25 (20–31)	27 (22–35)	24 (20–30)	**< 0.01** ^b^
Pulmonary capillary wedge pressure (mmHg)	8 (5–10)	8 (6–11)	8 (5–10)	0.494^b^
Cardiac index (L/min/m^2^)	3.1 (2.7–3.7)	3.1 (2.6–3.7)	3.1 (2.8–3.7)	0.191^b^
Pulmonary vascular resistance (WU)	2.8 (2.1–4.3)	3.4 (2.3–5.6)	2.7 (1.9–3.7)	**< 0.01** ^b^
Recipient comorbidities				
Hypertension	39 (16.5)	13 (15.9)	26 (16.8)	0.856^c^
Diabetes mellitus	30 (12.7)	8 (9.8)	22 (14.2)	0.329^c^
Coronary artery disease	24 (10.1)	9 (11.0)	15 (9.7)	0.753^c^
Other	5 (2.1)	2 (2.5)	3 (1.9)	0.787^c^
Reason for lung transplantation				
Interstitial lung diseases	121 (51.3)	44 (53.7)	77 (50.0)	0.593^c^
COPD	57 (24.1)	16 (19.5)	41 (26.5)	0.235^c^
Cystic fibrosis	40 (16.9)	12 (14.6)	28 (18.1)	0.503^c^
Sarcoidosis	8 (3.4)	4 (4.9)	4 (2.6)	0.353^c^
Pulmonary arterial hypertension	5 (2.1)	4 (4.9)	1 (0.6)	**0.031** ^c^
Other	6 (2.5)	2 (2.4)	4 (2.6)	0.948^c^
Transplant characteristics				
Waiting list time (days)	79 (25–301)	60 (21–355)	95 (26–288)	0.503^b^
Lung allocation score (points)	38 (35–43)	39 (36–43)	38 (35–43)	0.168^b^
pTLC ratio	1.06 (0.99–1.12)	1.06 (1.00–1.14)	1.06 (0.97–1.12)	0.593^b^
Undersized lungs (pTLC ratio < 0.9)	16 (6.8)	3 (3.7)	13 (8.4)	0.168^c^
Matched lungs (0.9 ≥ pTLC ratio ≤ 1.1)	140 (59.1)	51 (62.2)	89 (57.4)	0.478^c^
Oversized lungs (pTLC ratio > 1.1)	81 (34.2)	28 (34.1)	53 (34.2)	0.994^c^
Ischemic time left graft (hours)	6.9 (5.5–8.4)	6.7 (5.3–8.1)	6.9 (5.6–8.4)	0.451^b^
Ischemic time right graft (hours)	6.3 (5.3–7.6)	6.5 (5.3–7.8)	6.1 (5.3–7.5)	0.685^b^
Intubation before transplantation	2 (0.8)	1 (1.2)	1 (0.6)	0.646^c^
Preoperative ECMO	0 (0.0)	0 (0.0)	0 (0.0)	0.999^c^
Intraoperative ECMO	132 (55.5)	58 (70.7)	74 (47.7)	**< 0.01** ^c^
RBC transfusion	182 (76.8)	70 (85.4)	112 (72.3)	**0.023** ^c^
Number of RBCs during transplant	2 (1–5)	4 (2–6)	2 (0–4)	**< 0.01** ^b^
Donor parameters				
Age (years)	47 (33–59)	49 (37–62)	46 (30–58)	0.135^b^
Female gender	95 (40.1)	47 (57.3)	48 (31.9)	**< 0.01** ^c^
Height (m)	1.75 (1.70–1.81)	1.70 (1.65–1.80)	1.78 (1.70–1.83)	**< 0.01** ^b^
Weight (kg)	80 (70–87)	75 (65–86)	80 (70–90)	0.123^b^
Predicted body weight (kg)	71 (62–76)	63 (57–75)	73 (62–78)	**< 0.01** ^b^
Smoking history	70 (29.5)	27 (32.9)	43 (27.7)	0.406^c^
Antibiotics	182 (76.8)	66 (80.5)	116 (74.8)	0.328^c^
RBC transfusion	52 (21.9)	23 (28.0)	29 (18.7)	0.099^c^
Number of RBCs before transplant	0 (0–0)	0 (0–2)	0 (0–0)	0.070^b^
Duration of invasive ventilation (days)	3 (2–6)	3 (2–6)	3 (2–5)	0.072^b^
P/F ratio at the time of organ offer (mmHg)	429 (377–484)	431 (385–499)	429 (372–383)	0.751^b^
P_a_CO_2_ at the time of organ offer (mmHg)	40 (37–43)	41 (37–43)	40 (36–43)	0.428^b^

Continuous variables are presented as median (– interquartile range [IQR]); categorical variables are presented as numbers (%).

^a^ *p*-value for differences between lung transplant recipients with and without prolonged ventilation.

^b^ Mann-Whitney *U*-test.

^c^ Chi-squared test.

Abbreviations: PMV, prolonged mechanical ventilation; COPD, chronic obstructive pulmonary disease; pTLC ratio, predicted total lung capacity ratio; ECMO, extracorporeal membrane oxygenation; RBC, packed red blood cell transfusions; P/F ratio, the quotient of partial pressure of oxygen to the fractional inspired oxygen.

Significant *p*-values are in bold.

### Prolonged Mechanical Ventilation

PMV (>72 h) occurred in 82 patients (35%), with invasive ventilation totaling a median of 189 h [IQR 108–666 h] versus 32 h [21–45 h] in the non-PMV group (Table 2). In addition, patients were ventilated for more than 96 h and more than 7 days in 28% and 18%, respectively.

**TABLE 2:** Primary and secondary outcomes.

Primary and secondary outcomes	All patients (*n* = 237)	PMV (*n* = 82)	Non-PMV (*n* = 155)	*p*-value^a^
Primary outcome				
Duration of invasive ventilation (hours)	45.8 (24.8–109)	189 (108–666)	32.0 (20.7–45.1)	–
Secondary outcomes				
Admission to weaning readiness (hours)*	17.4 (11.4–27.4)	29.9 (17.6–47.4)	13.7 (9.0–20.4)	**< 0.01** ^b^
Extubation	212 (89.5)	57 (69.5)	155 (100.0)	**< 0.01** ^c^
Time to extubation (hours)	39.9 (23.3–65.0)	100 (78.0–132)	32.0 (20.9–45.0)	**< 0.01** ^c^
Non-invasive respiratory support^d^	95 (44.8)	35 (61.4)	60 (38.7)	**< 0.01** ^c^
NHFC	59 (24.9)	23 (28.0)	36 (23.2)	0.415^c^
NIV	83 (35.0)	31 (37.8)	52 (33.5)	0.514^c^
Duration of NIRS (hours)	50.5 (28.5–81.9)	48.8 (24.5–77.4)	55.9 (32.7–88.4)	0.517^b^
Reintubation	24 (10.1)	22 (39.3)	2 (1.3)	**< 0.01** ^c^
within < 72 h	14 (6.6)	13 (22.8)	1 (0.6)	**< 0.01** ^c^
Tracheostomy	39 (16.5)	39 (47.6)	0 (0.0)	**< 0.01** ^c^
Primary tracheostomy	25 (10.5)	25 (30.5)	0 (0.0)	**< 0.01** ^c^
ICU length of stay (days)	7 (5–15)	26 (12–46)	5 (4–8)	**< 0.01** ^b^
ICU mortality	2 (0.8)	1 (1.2)	1 (0.6)	0.646^c^

Continuous variables are presented as median (– interquartile range [IQR]); categorical variables are presented as numbers (%),

*The time between ICU admission (while patients were on pressure-controlled ventilation) and switch to pressure support ventilation, indicating “weaning readiness”.

^a^ *p-*value for differences between lung transplant recipients with and without prolonged ventilation.

^b^ Mann-Whitney *U*-test.

^c^ Chi-squared test.

^d^ NIV, and/or NHFC, use within 24 h following (first) extubation for at least 6 hours daily.

Abbreviations: PMV, prolonged mechanical ventilation; NHFC; nasal high flow cannula; NIV, non-invasive ventilation; NIRS, non-invasive respiratory support.

Significant *p*-values are in bold.

For PMV, the time between admission to the ICU and weaning readiness (as defined above) and the time to extubation was longer. A higher proportion of these patients also underwent primary tracheostomy, which resulted in fewer extubations. Among PMV subjects extubated, the percentage receiving non-invasive respiratory support was higher (61.4% versus 38.7%, *p* < 0.01). They also required more re-intubations (within a median of 48 h [32–90 h] following extubation) and tracheostomies (performed after a median of 11 days [7–14 days]), resulting in longer lengths of stay in the ICU (Table 2).

### Ventilatory Variables and Indexes

Overall, 2079 ventilatory variables with respective ABGs were collected over a median of 17.7 h [10.0–31.0 h] following ICU admission. Lung protective ventilation using low tidal volumes per recipients’ and donors’ PBW was provided to the PMV and non-PMV group (6.3 mL/kg [5.6–6.8 mL/kg] vs. 6.2 mL/kg [5.6–6.7 mL/kg], *p* = 0.409; and 5.7 mL/kg [5.1–6.7 mL/kg] vs. 5.8 mL/kg [5.2–6.4 mL/kg], *p* = 0.836).

PMV patients showed lower median P/F ratios (239 mmHg [184–308 mmHg] vs. 296 mmHg [254–345 mmHg], *p* < 0.01), higher ventilatory ratios (1.30 [1.17–1.50] vs. 1.18 [1.04–1.32], *p* < 0.01) and lower dynamic compliance (27 mL/cmH_2_O [22–31 mL/cmH_2_O] vs. 32 mL/cmH_2_O [28–38 mL/cmH_2_O], *p* < 0.01). Since minute ventilation was not different between groups, these patients also displayed higher MP (18.1 J/min [13.8–21.2 J/min] vs. 14.7 J/min [12.1–17.3 J/min], *p* < 0.01) owing to higher peak pressures. Consequently, power density was higher for PMV (e.g., LTC_dyn_-MP 7049 cmH_2_O^2^/min [5,255–8,299 cmH_2_O^2^/min] vs. 4,432 cmH_2_O^2^/min [3,781–5,889 cmH_2_O^2^/min], *p* < 0.01) (Table 3).

**TABLE 3:** Ventilatory variables and indexes following ICU admission.

Ventilatory variables	All patients (n = 237)	PMV (n = 82)	Non-PMV (n = 155)	*p*-value^a^
F_i_O_2_	39 (34–45)	41 (35–55)	38 (32–41)	**< 0.01** ^b^
Respiratory rate (1/min)	18 (15–20)	19 (17–20)	17 (15–19)	**< 0.01** ^b^
Tidal volume (mL)	402 (349–442)	382 (333–431)	408 (371–455)	**< 0.01** ^b^
Tidal volume/recipient PBW (mL/kg)	6.2 (5.6–6.8)	6.3 (5.6–6.8)	6.2 (5.6–6.7)	0.409^b^
Tidal volume/donor PBW (mL/kg)	5.9 (5.2–6.5)	5.7 (5.1–6.7)	5.8 (5.2–6.4)	0.836^b^
Minute ventilation (L/min)	6.9 (5.8–8.1)	7.2 (5.8–8.8)	6.8 (5.8–8.1)	0.253^b^
PEEP (cmH_2_O)	10 (8–10)	10 (9–12)	9 (8–10)	**< 0.01** ^b^
P_peak_ (cmH_2_O)	23 (21–25)	26 (23–28)	22 (20–24)	**< 0.01** ^b^
Dynamic driving pressure (cmH_2_O)	13 (12–15)	15 (13–16)	13 (11–14)	**< 0.01** ^b^
Arterial blood gas analysis				
P_a_O_2_ (mmHg)	104 (93–117)	99 (86–107)	108 (97–119)	**< 0.01** ^b^
P_a_CO_2_ (mmHg)	42 (38–46)	42 (38–46)	43 (38–47)	0.431^b^
pH	7.42 (7.38–7.45)	7.42 (7.38–7.44)	7.41 (7.37–7.45)	0.962^b^
Ventilatory indexes				
P/F ratio (mmHg)	278 (225–338)	239 (184–308)	296 (254–345)	**< 0.01** ^b^
Ventilatory ratio	1.21 (1.05–1.39)	1.30 (1.17–1.50)	1.18 (1.04–1.32)	**< 0.01** ^b^
LTC_dyn_ (mL/cmH_2_O)	31 (25–36)	27 (22–31)	32 (28–38)	**< 0.01** ^b^
Mechanical power (Joule/min)	15.3 (12.6–19.1)	18.1 (13.8–21.2)	14.7 (12.1–17.3)	**< 0.01** ^b^
PBW-MP (Joule/min/kg)	0.24 (0.20–0.29)	0.29 (0.23–0.35)	0.22 (0.19–0.26)	**< 0.01** ^b^
LTC_dyn_-MP (cmH_2_O^2^/min)	5,328 (3,999–7,092)	7,049 (5,255–8,299)	4,432 (3,781–5,889)	**< 0.01** ^b^

Continuous variables are presented as median (– interquartile range [IQR]); categorical variables are presented as numbers (%). After ICU admission, ventilatory variables and ABGs were collected over a median of 17.7 h [IQR, 10.0–31.0 h].

^a^ *p-*value for differences between lung transplant recipients with and without prolonged ventilation.

^b^ Mann-Whitney *U*-test.

^c^ Chi-squared test.

Abbreviations: PMV, prolonged mechanical ventilation; F_i_O_2_, fractional inspired oxygen; PBW, predicted body weight; PEEP, positive end-expiratory pressure; P/F ratio, the quotient of partial pressure of oxygen to the fractional inspired oxygen; LTC_dyn_, dynamic lung-thorax compliance; PBW-MP, mechanical power normalized to the predicted body weight; LTC_dyn_-MP, mechanical power normalized to the dynamic lung-thorax compliance.

Significant *p*-values are in bold.

**FIGURE 2:**
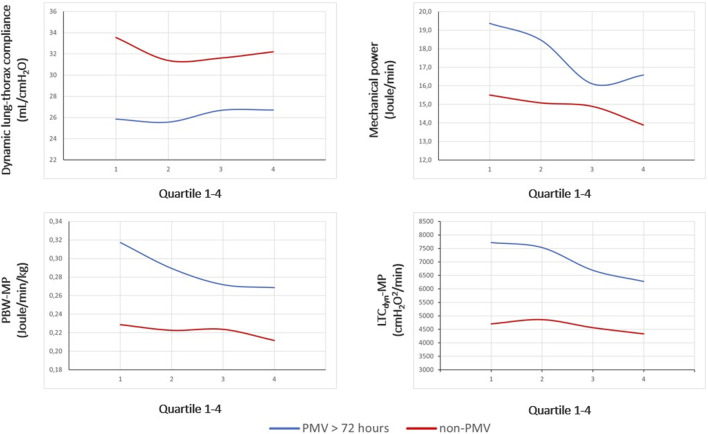
Between-group differences in trajectories of ventilatory indexes. Legend: Dynamic lung-thorax compliance, mechanical power, and power density are compared between PMV and non-PMV subjects according to the median of parameters within time quartiles collected following transplantation. Abbreviations*:* PBW-MP, mechanical power normalized to the predicted body weight; LTC_dyn_-MP, mechanical power normalized to dynamic lung-thorax compliance; PMV, prolonged mechanical ventilation.

Figure 2 illustrates the ventilatory variables of interest at different time points, stratified by group membership. According to the ANOVA, MP and power density declined consistently within both groups and similar to bivariate comparisons, there were significant differences in trajectories of variables between the PMV and non-PMV group following transplant (MP: *F*[1, 235] = 27.3, *p* < 0.01; LTC_dyn_-MP: *F*[1, 235] = 66.6, *p* < 0.01) (see Supplementary Tables S1, S2).

### Primary and Secondary Outcomes

**FIGURE 3:**
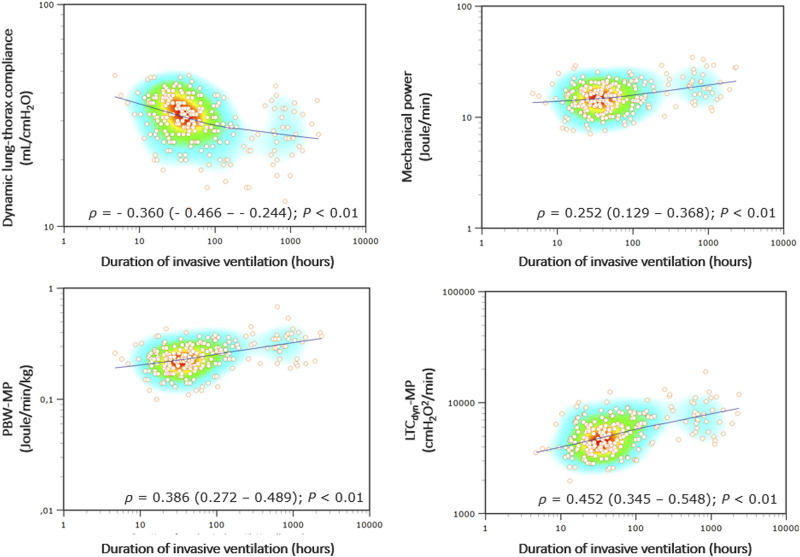
Correlations between ventilatory indexes and invasive ventilation duration. Legend: The heat map of Spearman’s correlation coefficients (*ρ*) with the LOESS (Local Regression Smoothing) trendline. The *Y* and *X*-axes have logarithmic scales. Abbreviations: *ρ*, Spearman’s correlation coefficient (with 95% confidence interval); PBW-MP, mechanical power normalized to the predicted body weight; LTC_dyn_-MP, mechanical power normalized to dynamic lung-thorax compliance.

**FIGURE 4:**
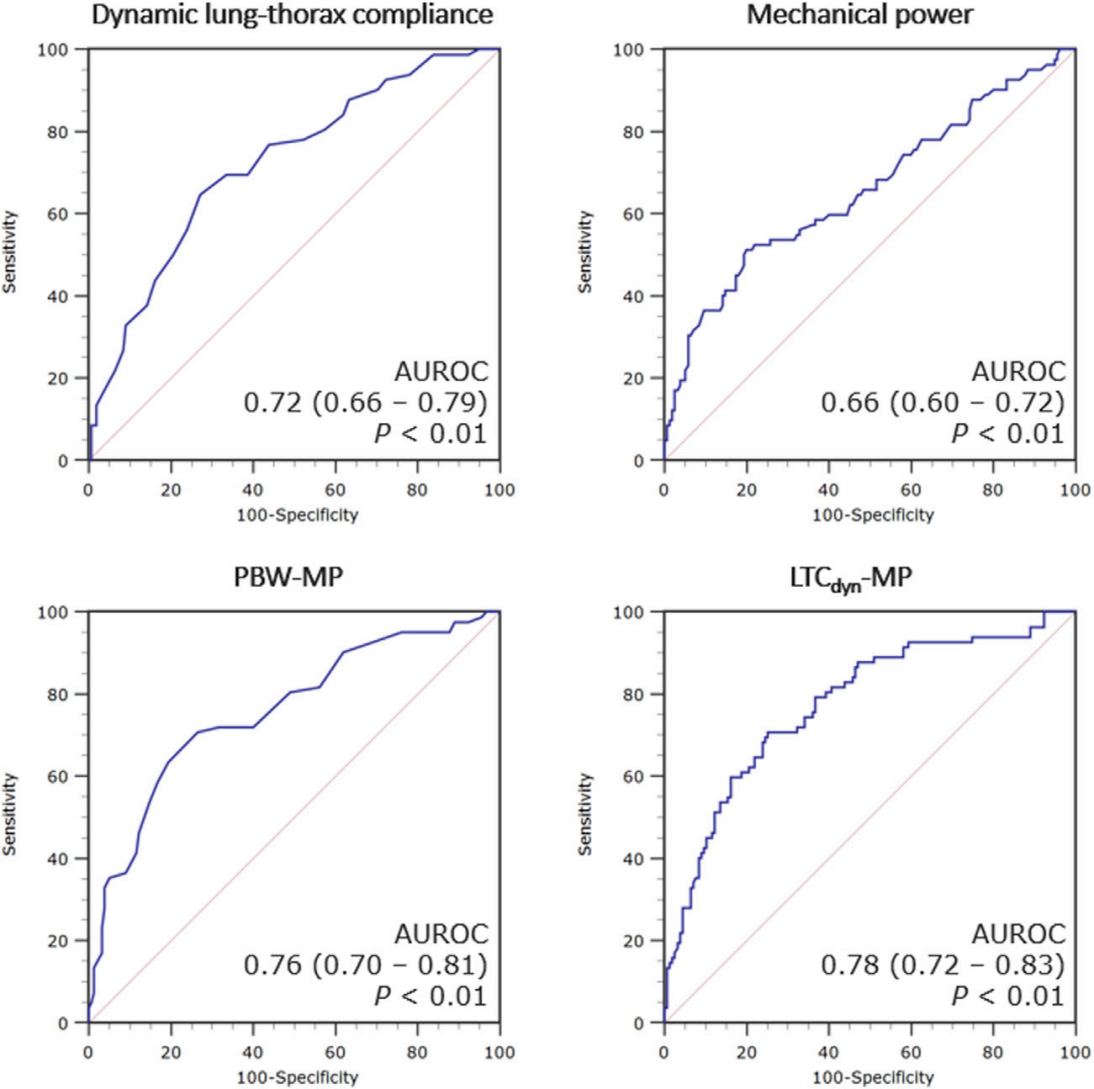
ROC curves for the ventilatory indexes predicting post-transplant prolonged mechanical ventilation. Legend: The accuracy of each index in predicting prolonged mechanical ventilation (>72 h) is presented as the area under the receiver operating characteristic (ROC) curve with 95% confidence intervals. Abbreviations: AUROC, area under the receiver operating characteristic curve; PBW-MP, mechanical power normalized to the predicted body weight; LTC_dyn_-MP, mechanical power normalized to dynamic lung-thorax compliance.

MP demonstrated a poor correlation with the duration of invasive ventilation (Spearmans’ coefficient 0.252 [95% CI 0.129–0.368]), which is consistently stronger with power density (*ρ* = 0.452 [0.345–0.548] for LTC_dyn_-MP) (Figure 3, see Supplementary Table S3). There was a poor diagnostic performance in predicting PMV for MP (AUROC 0.66 [95% CI 0.60–0.72], DOR 5.6, MCC 0.29), significantly increasing with power density (AUROC 0.78 [0.72–0.83], DOR 15.0, MCC 0.44 for LTC_dyn_-MP) (Figure 4; Table 4, see Supplementary Table S4; Supplementary Figure S2). The sensitivity analysis based on the 96-h and 7-day thresholds for defining prolonged ventilation or analyzing only 186 cases successfully extubated at the first attempt provided similar results (see Supplementary Tables S5–S8).

**TABLE 4:** Cross-validated performance of ventilatory indexes analyzed to predict post-transplant prolonged mechanical ventilation.

Ventilatory indexes (threshold)	Sensitivity	Specificity	PPV	NPV	Accuracy	PLR	NLR	DOR	F_1_	MCC
P/F ratio (251 mmHg)	54 (29–78)	71 (53–85)	52 (33–69)	73 (61–83)	65 (51–78)	2.5 (1.1–5.8)	0.8 (1.3–0.5)	5.5	0.53	0.25
Ventilatory ratio (1.21)	56 (32–78)	58 (39–75)	41 (28–57)	72 (57–83)	58 (42–72)	1.4 (0.7–2.6)	0.7 (1.4–0.4)	2.5	0.46	0.14
LTC_dyn_ (28 mL/cmH_2_O)	63 (41–83)	74 (56–88)	57 (39–72)	79 (66–88)	70 (55–82)	2.7 (1.3–5.4)	0.5 (1.0–0.3)	6.3	0.59	0.37
Mechanical power (18.1 J/min)	48 (23–73)	80 (62–91)	58 (35–75)	74 (63–82)	69 (54–81)	3.4 (1.1–14)	0.7 (1.1–0.4)	5.6	0.51	0.29
PBW-MP (0.26 J/min/kg)	69 (42–88)	73 (54–87)	58 (41–72)	81 (67–90)	71 (56–83)	3.0 (1.4–6.6)	0.4 (0.9–0.2)	8.6	0.62	0.40
LTC_dyn_-MP (5,844 cmH_2_O^2^/min)	71 (44–90)	74 (56–87)	61 (43–74)	82 (68–87)	73 (58–84)	4.6 (1.6–19)	0.4 (0.9–0.3)	15.0	0.65	0.44

Results of 2-times repeated, 5-fold cross-validation. Mean metrics of diagnostic accuracy (with 95% confidence intervals) based on threshold values associated with the *Youden index* (presented as the mean of the thresholds derived from the training sets).

Abbreviations: PPV, positive predictive value; NPV, negative predictive value; PLR, positive likelihood ratio; NLR, negative likelihood ratio; DOR, diagnostic odds ratio; F_1_, F_1_ score; MCC, Matthews’ correlation coefficient; P/F ratio, the ratio of partial pressure of oxygen to fractional inspired oxygen; VR, ventilatory ratio; LTC_dyn_, dynamic lung-thorax compliance; PBW-MP, mechanical power normalized to the predicted body weight; LTC_dyn_-MP, mechanical power normalized to dynamic lung-thorax compliance.

Patients were stratified by whether they had received post-extubation NHFC/NIV and compared to patients with primary tracheostomy. The respiratory mechanics of these three groups differed significantly, with tracheotomized patients demonstrating less favorable mechanics and outcomes (e.g., lower dynamic compliance, higher power density, and a longer duration of invasive ventilation). Similarly, extubated patients requiring non-invasive respiratory support showed higher power densities than those receiving pure oxygen insufflation. Re-intubations and tracheostomies were also significantly higher (see Supplementary Table S9).

### Binary Logistic Regression Analysis Results

In univariable analysis, MP and power density (LTCdyn-MP) were independently related to PMV. After adjusting for recipient, transplant, and donor characteristics, only power density (OR 1.54 per cmH_2_O^2^/min * 10^−3^ [95% CI 1.30–1.83]; *p* < 0.01) remained in the multivariable model, along with female gender (OR 2.45 [95% CI 1.29–4.66]; *p* < 0.01), recipients’ mean pulmonary artery pressure before transplant (OR 1.05 mmHg^−1^ [95% CI 1.02–1.09]; *p* < 0.01), and P/F ratio after transplant (OR 0.57 mmHg^−1^ * 10^−2^ [95% CI 0.36–0.88]; *p* = 0.012) (see Supplementary Tables S10, S11).

### PGD at T+72 h After Transplant

There were 18 out of 237 patients with PGD grade 3 (7.6%), 28 patients with PGD grade 2 (11.8%), and 11 patients with PGD grade 1 (4.6%). Moreover, we identified a subgroup of 22 (9.3%) mechanically ventilated patients without PGD having a similar duration of mechanical ventilation, P/F ratios, MP, and power density levels. The patients with PGD grade 3 compared with grades 1/2 had longer durations of invasive ventilation and fewer extubations, despite no significant differences in baseline characteristics, donor parameters, or ventilatory indexes (see Supplementary Table S12).

### Exploratory Outcomes

PMV was more prevalent in females; they also experienced more re-intubations and tracheostomies, and respiratory mechanics significantly differed from male recipients. Females demonstrated lower tidal volumes and higher airway pressures (along with lower dynamic compliance), but MP was higher in males owing to substantially higher tidal volumes (despite lower airway pressure). After normalizing MP to dynamic compliance, females exhibited significantly higher power density levels (see Supplementary Table S13).

## Discussion

Study results can be summarized as follows: Prolonged mechanical ventilation (>72 h) following double lung transplantation without postoperative extracorporeal support occurred in 35% of cases. More severe impairment in respiratory mechanics, ventilation efficiency, and gas exchange was observed among PMV patients, associated with less favorable clinical outcomes, such as increased re-intubation and tracheostomy rates. MP assessed in the early postoperative period significantly and independently correlated with post-transplant invasive ventilation, with power density demonstrating stronger correlations and a more accurate prediction of PMV. Patients requiring post-extubation non-invasive respiratory support and those experiencing primary tracheostomy had more compromised respiratory mechanics resulting in higher power density and inferior outcomes.

A few studies have examined PMV after lung transplantation, reporting frequencies of 5%–28% with a wide range of definitions ranging from 72 h to 60 days [4, 5, 28, 29]. Previous reports showed higher re-intubation and tracheostomy rates among PMV patients, translating into longer ICU lengths of stay [30, 31] and matching our results. Moreover, although not explicitly demonstrated in lung transplant recipients, post-extubation respiratory failure requiring re-intubation involves significant morbidity and mortality [32]. Notably, an early approach to tracheostomy (<3 days) may enhance postoperative recovery after lung transplant, resulting in shorter ventilation times and ICU lengths of stay [33]. Tracheostomy was performed after a median of 11 days in the present analysis, and these patients showed the highest power density levels, which allows for accurate separation from non-tracheotomized patients. This way, these indexes can assist in preventing delays in the decision-making process before tracheostomy.

Knowing whether a patient will require PMV is critical due to its association with inferior outcomes. Numerous independent predictors have been identified, including renal replacement therapy, anastomotic dehiscence, and neurological complications [5]. However, these factors are most often related to complications resulting from surgery rather than pertaining to lung allograft function. In contrast, the ventilatory variables used for MP calculation reflect patients’ respiratory mechanics. In the present analysis, power density (mechanical stress intensity) outperformed mechanical power in correlating with invasive ventilation duration and predicting PMV. Power density (e.g., LTC_dyn_-MP) mainly depends on airway pressure and respiratory rate, and current data suggest that these parameters are most relevant in determining whether a patient will require a prolonged course of ventilation [4, 29] or even be unable to wean off the ventilator [7, 34]. Accordingly, in the present analysis, airway pressures and respiratory rate but not PBW-adjusted tidal volumes significantly differed between PMV and non-PMV subjects. Similarly, Thakuria et al. found that mechanical ventilation with high inflation pressure, irrespective of the related tidal volume, resulted in poor physiological and clinical outcomes after lung transplant (e.g., reduced 6 month survival) [10].

Specifically, these observations may be relevant to undersized allografts characterized by a low pTLC ratio [35]. Tidal volumes adjusted to the recipients’ PBW will result in potentially injurious inflation pressures, contributing to the development of ventilator-induced lung injury [13], PGD [15], and PMV [4, 14]. However, neither the pTLC ratios nor the percentages of patients with undersized allografts varied between groups in the present analysis. Patients in the PMV group, despite receiving low tidal volume ventilation, experienced higher peak airway pressure, lowered dynamic compliance (indicating a lower ventilated lung volume), and higher power density. A similar finding was made in the subgroup of female recipients, independently associated with higher PMV rates. Nevertheless, as the study is observational, no cause-and-effect relationship can be drawn between high airway pressure (and power density), lung injury, and PMV. It should be noted, however, that 75% of PMV patients experienced peak airway pressures below 28 cmH_2_O, which is considered within safe limits [21, 36, 37].

Several clinical, transplant, and donor characteristics differed significantly among PMV patients. This group received more red cell transfusions during surgery, associated with a higher risk of PGD [38, 39]. In addition, higher mean pulmonary artery pressures and a diagnosis of pulmonary arterial hypertension, well-known risk factors for left ventricular dysfunction following lung transplantation [38, 40], as well as a greater proportion of recipients receiving intraoperative ECMO may indicate more severe pulmonary edema, which adversely affects early graft function and delays ventilator weaning and time to extubation [3]. Interestingly, according to the logistic regression analysis, neither red cell transfusions nor ECMO procedures were retained in the final multivariable PMV prediction model.

Basically, the MP concept was introduced to shed further light on the mechanisms involved in ventilator-induced lung injury, as it converges all ventilatory variables deemed responsible [6]. However, MP required to maintain sufficient oxygenation and decarboxylation during controlled ventilation may correlate with the respiratory muscles’ workload during spontaneous breathing. Indeed, MP and weaning failure have been linked in previous studies, with power density showing consistently higher predictive ability [7, 8]. Similarly, an observational study showed that higher MP was independently associated with postoperative respiratory failure requiring re-intubation in non-cardiac surgery patients [41]. Furthermore, spontaneous breathing power, determined by esophageal pressure measurement, accurately predicted respiratory treatment escalation in COVID-19 pneumonia patients [42]. There is a misconception, however, that a higher MP always indicates inferior clinical outcomes (e.g., weaning failure related to impaired respiratory mechanics). Since iso-MP may be derived from low pressures and high tidal volumes (equal to elevated compliance resulting in low power density) or high pressures coupled with low tidal volumes (consistent with low compliance and high power density), the latter condition relates to respiratory mechanics that are more prone to weaning failure [7, 8]. Accordingly, exploratory analyses revealed that males were less likely to require PMV despite higher MP, demonstrating significantly lower power density (owing to higher dynamic compliance) than female recipients. Given that MP is thought to significantly contribute to ventilator-induced lung injury [6, 43], the question arises whether MP is a cause or correlate of impaired respiratory mechanics associated with inferior outcomes [44].

### Limitations

This study has limitations. First, nearly half of the patients screened were excluded, mainly due to single lung transplantation and requiring ECMO after surgery, resulting in a highly selected group of patients and limiting the generalizability of the results. However, given that both conditions impact either the respiratory systems’ mechanical properties or pulmonary gas exchange, this step was necessary to determine whether there is a relationship between early graft function, MP, and PMV. Second, although we performed cross-validation, it is uncertain whether the results apply to other centers due to the study`s retrospective nature, monocentric design, and lack of an external validation group. Third, ventilatory variables were recorded during pressure-controlled ventilation, but we cannot rule out that some patients already had spontaneous breathing activity, affecting measurements of some variables (e.g., tidal volume) and consequently distorting values for dynamic compliance and MP. Fourth, since this was a retrospective analysis restricting the available ventilatory variables, we used a simplified equation for MP calculation [20], and correlations and predictions of PMV might have been more accurate with a more comprehensive formula [45]. Fifth, we did not assess primary graft dysfunction systematically at the designated time points T0, T+24, T+48, and T+72 [2]. However, evidence suggests that PGD scores are ineffective at predicting PMV [4], which might be related to difficulties in adequately grading PGD and the fact that scoring does not provide a physiological assessment of the allograft [46].

### Conclusion

In double lung transplant recipients without postoperative extracorporeal support, PMV was observed in 35% of cases and related to impairments in respiratory mechanics and pulmonary gas exchange. A significant correlation was found between MP and the duration of post-transplant invasive ventilation, with power density showing stronger correlations and predicting PMV more accurately. These indexes may assist clinicians in identifying patients at risk for PMV, which is associated with inferior outcomes such as increased re-intubation and tracheostomy rates. Clinical strategies may change in the event of persistently high power density after transplant, such as providing early tracheostomy or prophylactic non-invasive respiratory support following extubation. Findings need to be confirmed in future studies and whether differences in MP may ultimately impact long-term clinical outcomes after lung transplantation.

## Data Availability

The raw data supporting the conclusion of this article will be made available by the authors, without undue reservation.

## Abbreviations

95% CI, 95% confidence interval; ABGs, Arterial blood gas analyses; AUROC, Area under the receiver operating characteristic curve; CPAP, Continuous positive airway pressure; DOR, Diagnostic odds ratio; ECMO, Extracorporeal membrane oxygenation; ISHLT, International Society of heart and lung transplantation; LTC_dyn_, Dynamic lung-thorax compliance (equals the respiratory system compliance); LTC_dyn_-MP, Mechanical power normalized to the dynamic lung-thorax compliance; MCC, Matthews’ correlation coefficient; MP, Mechanical power; NHFC, Nasal high-flow cannula; NIV, Non-invasive ventilation; OR, Odds ratio; ∆P_aw_, Dynamic driving pressure; PBW, Predicted body weight; PBW-MP, Mechanical power normalized to the predicted body weight; PEEP, Positive end-expiratory pressure; P/F ratio, Quotient of partial pressure of oxygen to fractional inspired oxygen; PGD, Primary graft dysfunction; PMV, Prolonged mechanical ventilation; P_peak_, Peak airway pressure; pTLC ratio, predicted donor/recipient total lung capacity ratio; RBC, Packed red blood cells; VR, Ventilatory ratio.

## References

[1] Clausen E Cantu E. Primary Graft Dysfunction: What We Know. J Thorac Dis (2021) 13(11):6618–27. 10.21037/jtd-2021-18 34992840 PMC8662499

[2] Snell GI Yusen RD Weill D Strueber M Garrity E Reed A Report of the ISHLT Working Group on Primary Lung Graft Dysfunction, Part I: Definition and Grading – A 2016 Consensus Group Statement of the International Society for Heart and Lung Transplantation. J Heart Lung Transpl (2017) 36(10):1097–103. 10.1016/j.healun.2017.07.021 28942784

[3] Sage AT Peel J Valero J Yeung JC Mingyao L Cypel M Time to Extubation for Lung Transplant Recipients Represents a Pragmatic Endpoint to Guide the Development of Prognostic Tests. J Heart Lung Transpl (2023) S1053-2498(23):01926–5. Online ahead of print. 10.1016/j.healun.2023.06.019 37406839

[4] Schwarz S Benazzo A Dunkler D Muckenhuber M Sorbo LD Di Nardo M Ventilation Parameters and Early Graft Function in Double Lung Transplantation. J Heart Lung Transpl (2021) 40(1):4–11. 10.1016/j.healun.2020.10.003 33144029

[5] Hadem J Gottlieb J Seifert D Fegbeutel C Sommer W Greer M Prolonged Mechanical Ventilation After Lung Transplantation – A Single-Center Study. Am J Transpl (2016) 16(5):1579–87. 10.1111/ajt.13632 26607844

[6] Gattinoni L Tonetti T Cressoni M Cadringher P Herrmann P Moerer O Ventilator-Related Causes of Lung Injury: The Mechanical Power. Intensive Care Med (2016) 42(10):1567–75. 10.1007/s00134-016-4505-2 27620287

[7] Yan Y Xie Y Chen X Sun Y Du Z Wang Y Mechanical Power Is Associated with Weaning Outcome in Critically Ill Mechanically Ventilated Patients. Sci Rep (2022) 12(1):19634. 10.1038/s41598-022-21609-2 36385129 PMC9669041

[8] Ghiani A Paderewska J Walcher S Tsitouras K Neurohr C Kneidinger N. Mechanical Power Normalized to Lung-Thorax Compliance Indicates Weaning Readiness in Prolonged Ventilated Patients. Sci Rep (2022) 12(1):6. 10.1038/s41598-021-03960-y 34997005 PMC8741981

[9] De Perrot M Imai Y Volgyesi GA Waddell TK Liu M Mullen JB Effect of Ventilator-Induced Lung Injury on the Development of Reperfusion Injury in a Rat Lung Transplant Model. J Thorac Cardiovasc Surg (2002) 124(6):1137–44. 10.1067/mtc.2002.125056 12447179

[10] Thakuria L Davey R Romano R Carby MR Kaul S Griffiths MJ Mechanical Ventilation After Lung Transplantation. J Crit Care (2016) 31(1):110–8. 10.1016/j.jcrc.2015.09.021 26590855

[11] Verbeek GL Myles PS Westall GP Lin E Hastings SL Marasco SF Intra-Operative Protective Mechanical Ventilation in Lung Transplantation: A Randomised, Controlled Trial. Anesthesia (2017) 72(8):993–1004. 10.1111/anae.13964 28695586

[12] Currey J Pilcher DV Davies A Scheinkestel C Botti M Bailey M Implementation of a Management Guideline Aimed at Minimizing the Severity of Primary Graft Dysfunction After Lung Transplant. J Thorac Cardiovasc Surg (2010) 139(1):154–61. 10.1016/j.jtcvs.2009.08.031 19909995

[13] Kozower BD Meyers BF Ciccone AM Guthrie TJ Patterson GA. Potential for Detrimental Hyperinflation After Lung Transplantation With Application of Negative Pleural Pressure to Undersized Lung Grafts. J Thorac Cardiovasc Surg (2003) 125(2):430–2. 10.1067/mtc.2003.139 12579123

[14] Dezube R Arnaoutakis GJ Reed RM Bolukbas S Shah AS Orens JB The Effect of Lung-Size Mismatch on Mechanical Ventilation Tidal Volumes After Bilateral Lung Transplantation. Interact Cardiovasc Thorac Surg (2013) 16(3):275–81. 10.1093/icvts/ivs493 23243035 PMC3568811

[15] Tague LK Bedair B Witt C Byers DE Vazquez-Guillamet R Kulkarni H Lung Protective Ventilation Based on Donor Size Is Associated with A Lower Risk of Severe Primary Graft Dysfunction After Lung Transplantation. J Heart Lung Transpl (2021) 40(10):1212–1222. 10.1016/j.healun.2021.06.016 PMC8886387 34353713

[16] Di Nardo M Tikkanen J Husain S Singer LG Cypel M Ferguson ND Postoperative Management of Lung Transplant Recipients in the Intensive Care Unit. Anesthesiology (2022) 136(3):482–499. 10.1097/ALN.0000000000004054 34910811

[17] Rocco M Conti G Antonelli M Bufi M Costa MG Alampi D Non-Invasive Pressure Support Ventilation in Patients With Acute Respiratory Failure After Bilateral Lung Transplantation. Intensive Care Med (2001) 27(10):1622–6. 10.1007/s001340101063 11685303

[18] Stocks J Quanjer PH. Reference Values for Residual Volume, Functional Residual Capacity and Total Lung Capacity. ATS Workshop on Lung Volume Measurements. Official Statement of the European Respiratory Society. Eur Respir J (1995) 8(3):492–506. 10.1183/09031936.95.08030492 7789503

[19] Sinha P Fauvel NJ Singh P Soni N. Analysis of Ventilatory Ratio as a Novel Method to Monitor Ventilatory Adequacy at the Bedside. Crit Care (2013) 17:R34. 10.1186/cc12541 23445563 PMC4057449

[20] Becher T van der Staay M Schädler D Frerichs I Weiler N. Calculation of Mechanical Power for Pressure-Controlled Ventilation. Intensive Care Med (2019) 45(9):1321–3. 10.1007/s00134-019-05636-8 31101961

[21] Brower RG Matthay MA Morris A Schoenfeld D Thompson BT Wheeler A Ventilation with Lower Tidal Volumes as Compared with Traditional Tidal Volumes for Acute Lung Injury and the Acute Respiratory Distress Syndrome. N Engl J Med (2000) 342(18):1301–1308. 10.1056/NEJM200005043421801 10793162

[22] Marini JJ Rocco PRM Gattinoni L. Static and Dynamic Contributors to Ventilator-Induced Lung Injury in Clinical Practice. Pressure, Energy, and Power. Am J Respir Crit Care Med (2020) 201(7):767–74. 10.1164/rccm.201908-1545CI 31665612 PMC7124710

[23] Faffe DS Zin WA. Lung Parenchymal Mechanics in Health and Disease. Physiol Rev (2009) 89(3):759–75. 10.1152/physrev.00019.2007 19584312 PMC7203567

[24] Beer A Reed RM Bölükbas S Budev M Chaux G Zamora MR Mechanical Ventilation After Lung Transplantation. An International Survey of Practices and Preferences. Ann Am Thorac Soc (2014) 11(4):546–53. 10.1513/AnnalsATS.201312-419OC 24640938

[25] DeLong ER DeLong DM Clarke-Pearson DL. Comparing the Areas Under Two or More Correlated Receiver Operating Characteristic Curves: A Nonparametric Approach. Biometrics (1988) 44:837–845. 10.2307/2531595 3203132

[26] Chicco D Jurman G. The Advantages of the Matthews Correlation Coefficient (MCC) Over F1 Score and Accuracy in Binary Classification Evaluation. BMC Genomics (2020) 21:6. 10.1186/s12864-019-6413-7 31898477 PMC6941312

[27] Thabane L Mbuagbaw L Zhang S Samaan Z Marcucci M Ye C A Tutorial on Sensitivity Analyses in Clinical Trials: The What, Why, When and How. BMC Med Res Methodol (2013) 13:92. 10.1186/1471-2288-13-92 23855337 PMC3720188

[28] Atchade E Boughaba A Tran Dinh A Jean-Baptiste S Tanaka S Copelovici L Prolonged Mechanical Ventilation After Lung Transplantation: Risks Factors and Consequences on Recipient Outcome. Front Med (Lausanne) (2023) 10:1160621. 10.3389/fmed.2023.1160621 37228395 PMC10203407

[29] Benazzo A Schwarz S Frommlet F Sinn K Schweiger T Klikovits T Donor Ventilation Parameters as Predictors for Length of Mechanical Ventilation After Lung Transplantation: Results of a Prospective Multicenter Study. J Heart Lung Transpl (2021) 40(1):33–41. 10.1016/j.healun.2020.10.008 33246712

[30] Padia SA Borja MC Orens JB Yang SC Jhaveri RM Conte JV. Tracheostomy Following Lung Transplantation Predictors and Outcomes. Am J Transpl (2003) 3(7):891–5. 10.1034/j.1600-6143.2003.00170.x 12814482

[31] Feltracco P Milevoj M Alberti V Carollo C Michieletto E Rea F Early Tracheostomy Following Lung Transplantation. Transpl Proc (2011) 43(4):1151–5. 10.1016/j.transproceed.2011.01.154 21620075

[32] Epstein SK Ciubotaru RL Wong JB. Effect of Failed Extubation on the Outcome of Mechanical Ventilation. Chest (1997) 112(1):186–92. 10.1378/chest.112.1.186 9228375

[33] Miyoshi R Chen-Yoshikawa TF Hamaji M Kawaguchi A Kayawake H Hijiya K Effect of Early Tracheostomy on Clinical Outcomes in Critically Ill Lung Transplant Recipients. Gen Thorac Cardiovasc Surg (2018) 66(9):529–36. 10.1007/s11748-018-0949-3 29796751

[34] Pham T Heunks L Bellani G Madotto F Aragao I Beduneau G Weaning From Mechanical Ventilation in Intensive Care Units Across 50 Countries (Wean Safe): A Multicentre, Prospective, Observational Cohort Study. Lancet Respir Med (2023) 11(5):465–476. 10.1016/S2213-2600(22)00449-0 36693401

[35] Eberlein M Reed RM Bolukbas S Diamond JM Wille KM Orens JB Lung Size Mismatch and Primary Graft Dysfunction After Bilateral Lung Transplantation. J Heart Lung Transpl (2015) 34(2):233–240. 10.1016/j.healun.2014.09.030 PMC4329253 25447586

[36] Goligher EC Costa ELV Yarnell CJ Brochard LJ Stewart TE Tomlinson G Effect of Lowering VT on Mortality in Acute Respiratory Distress Syndrome Varies With Respiratory System Elastance. Am J Respir Crit Care Med (2021) 203(11):1378–1385. 10.1164/rccm.202009-3536OC 33439781

[37] Tobin MJ. The Dethroning of 6 mL/kg as the “Go-To” Setting in Acute Respiratory Distress Syndrome. Am J Respir Crit Care Med (2021) 204(7):868–869. 10.1164/rccm.202105-1320LE 34186011 PMC8528533

[38] Diamond JM Lee JC Kawur SM Shah RJ Localio AR Bellamy SL Clinical Risk Factors for Primary Graft Dysfunction After Lung Transplantation. Am J Respir Crit Care Med (2013) 187(5):527–534. 10.1164/rccm.201210-1865OC 23306540 PMC3733407

[39] Subramaniam K Loor G Chan E Bottiger B Ius F Hartwig M Intraoperative Red Blood Cell Transfusion and Primary Graft Dysfunction After Lung Transplantation. Transplantation (2023) 107(7):1573–1579. 10.1097/TP.0000000000004545 36959119

[40] Kortchinsky T Mussot S Rezaiguia S Artiguenave M Fadel E Stephan F. Extracorporeal Life Support in Lung and Heart-Lung Transplantation for Pulmonary Hypertension in Adults. Clin Transpl (2016) 30(9):1152–8. 10.1111/ctr.12805 27412378

[41] Santer P Wachtendorf LJ Suleiman A Houle TT Fassbender P Costa EL Mechanical Power during General Anesthesia and Postoperative Respiratory Failure: A Multicenter Retrospective Cohort Study. Anesthesiology (2022) 137(1):41–54. 10.1097/ALN.0000000000004256 35475882

[42] Gattarello S Coppola S Chiodaroli S Pozzi T Camporota L Saager L Mechanical Power Ratio and Respiratory Treatment Escalation in COVID-19 Pneumonia: A Secondary Analysis of A Prospectively Enrolled Cohort. Anesthesiology (2023) 138(3):289–298. 10.1097/ALN.0000000000004465 36571571 PMC9904389

[43] Serpa Neto A Deliberato RO Johnson AEW Bos LD Amorim P Pereira SM Mechanical Power of Ventilation Is Associated with Mortality in Critically Ill Patients: An Analysis of Patients in Two Observational Cohorts. Intensive Care Med (2018) 44(11):1914–1922. 10.1007/s00134-018-5375-6 30291378

[44] Gama de Abreu M Sessler DI. Mechanical Power: Correlate or Cause of Ventilator-Induced Lung Injury? Anesthesiology (2022) 137(1):6–8. 10.1097/ALN.0000000000004240 35560172

[45] Trinkle CA Broaddus RN Sturgill JL Waters CM Morris PE. Simple, Accurate Calculation of Mechanical Power in Pressure-Controlled Ventilation (PCV). Intensive Care Med Exp (2022) 10(1):22. 10.1186/s40635-022-00448-5 35644896 PMC9148680

[46] Schwarz S Muckenhuber M Benazzo A Beer L Gittler F Prosch H Interobserver Variability Impairs Radiologic Grading of Primary Graft Dysfunction After Lung Transplantation. J Thorac Cardiovasc Surg (2019) 158(3):955–62. 10.1016/j.jtcvs.2019.02.134 31204131

