# Supplementary material for: Mechanical Power Density Predicts Prolonged Ventilation Following Double Lung Transplantation
Source: Transpl Int. 2023 Sep 20;36:11506. doi: 10.3389/ti.2023.11506 (PMC10548550; doi:10.3389/ti.2023.11506)
Supplement: Supplementary file 1 [file DataSheet1.PDF]

Mechanical power density predicts prolonged ventilation following double lung transplantation

Alessandro Ghiani, MD; Nikolaus Kneidinger, MD; Claus Neurohr, MD; Sandra Frank, MD; Ludwig Christian Hinske, MD; Christian Schneider, MD; Sebastian Michel, MD; and Michael Irlbeck, MD.

Supplementary materials

- Definitions of ventilatory indexes and mechanical power
- **Figure S1:** Trajectories of invasive mechanical ventilation after lung transplantation
- **Table S1:** Results of repeated measures ANOVA – within-subjects time effects
- **Table S2:** Results of repeated measures ANOVA – between-group differences
- **Table S3:** Correlations between ventilatory indexes and post-transplant invasive ventilation duration
- **Table S4:** Area under the ROC curve of ventilatory indexes regarding the prediction of post-transplant prolonged mechanical ventilation (> 72 hours)
- **Figure S2:** Comparison of ROC curves for mechanical power and power density predicting post-transplant prolonged mechanical ventilation (> 72 hours)
- **Table S5:** Sensitivity analysis: Area under the ROC curve of ventilatory indexes regarding the prediction of post-transplant invasive mechanical ventilation > 96 hours
- **Table S6:** Sensitivity analysis: Area under the ROC curve of ventilatory indexes regarding the prediction of post-transplant invasive mechanical ventilation > 7 days
- **Table S7:** Sensitivity analysis: Correlations between ventilatory indexes and post-transplant invasive ventilation duration in patients successfully extubated at the first attempt (N = 186)
- **Table S8:** Sensitivity analysis: Area under the ROC curve of ventilatory indexes regarding the prediction of post-transplant prolonged mechanical ventilation (> 72 hours) in patients successfully extubated at the first attempt (N = 186)
- **Table S9:** Comparison of lung transplant recipients with different levels of respiratory support
- **Table S10:** Variables independently associated with prolonged mechanical ventilation following double lung transplantation – Results of univariable and multivariable binary logistic regression analysis
- **Table S11:** Multivariable binary logistic regression model
- **Table S12:** Comparison of patients with different primary graft dysfunction (PGD) grades at time-point T+72h after lung transplant
- **Table S13:** Comparison of female and male lung transplant recipients
- **References**

Definitions of ventilatory indexes and mechanical power

**Ventilatory ratio (VR)**	Ventilatory ratio (VR) is a surrogate of pulmonary dead space fraction and a simple bedside index of impaired efficiency of ventilation[1-2]:<br><br>$$\mathbf{VR = VE_{measured} * P_aCO_{2\text{-}measured} / VE_{predicted} * P_aCO_{2\text{-}ideal}}$$<br><br>$VE_{measured}$ is the measured minute ventilation (mL/min), $P_aCO_{2\text{-}measured}$ is the measured arterial pressure of carbon dioxide (mmHg), $VE_{predicted}$ is the predicted minute ventilation calculated as predicted bodyweight x 100 (mL/min), and P_aCO2_{ideal} is the expected arterial pressure of carbon dioxide in normal lungs if ventilated with the predicted minute ventilation. $P_aCO_{2\text{-}ideal}$ is set at 37.5 mmHg (5 kPa) for all patients. VR is a unitless ratio, with a value approximating one representing normal ventilating lungs.
**Mechanical power (MP)**	Mechanical power (MP)[3] provided by the ventilator in the pressure-controlled mode was calculated using the simplified formula proposed by Becher et al., including VT, respiratory rate (RR), and P_{peak}[4-5]:<br><br>$$\mathbf{MP\ (Joule/min) = 0.098 * VT * RR * P_{peak}}$$<br>$$\mathbf{MP\ (Joule/min) = 0.098 * VE * P_{peak}}$$<br><br>With each breath the ventilator delivers, a certain amount of energy (Joule) is transferred to the patient's respiratory system. This energy is mainly used to overcome the airways' resistance, inflate the lungs, and expand the thoracic cage.
**Mechanical power normalized to dynamic lung-thorax compliance (LTC_{dyn}-MP)**	MP normalized to dynamic lung-thorax compliance (LTC_{dyn}-MP; a measure of *"mechanical power density"*, which equals the intensity of mechanical stress exerted on the respiratory system) was calculated using MP and dynamic lung-thorax compliance (LTC_{dyn})[6]:<br><br>$$\mathbf{LTC_{dyn}\text{-}MP\ (J/min * cmH_2O/mL) = MP / LTC_{dyn}}$$<br>$$\mathbf{LTC_{dyn}\text{-}MP\ (J/min * cmH_2O/mL) = (0.098 * VT * RR * P_{peak}) * (\Delta P_{aw} / VT)}$$<br>$$\downarrow$$<br>$$\mathbf{LTC_{dyn}\text{-}MP\ (cmH_2O^2/min) = RR * P_{peak} * \Delta P_{aw}}$$<br>$$\mathbf{LTC_{dyn}\text{-}MP\ (cmH_2O^2/min) = RR * P_{peak} * (P_{peak} - PEEP)}$$<br><br>Generally, mechanical power density measures how much energy is transferred per unit volume displacement in a given period. The formula accounts for different effects of changes in respiratory rate, inspiratory pressure (P_{peak}), and PEEP (and thus changes in dynamic driving pressure = ΔP_{aw}) on delivered power density. Increasing RR leads to a linear rise in stress intensity, while increasing pressure results in an exponential increment in stress intensity[3].

Figure S1. Trajectories of invasive mechanical ventilation after lung transplantation

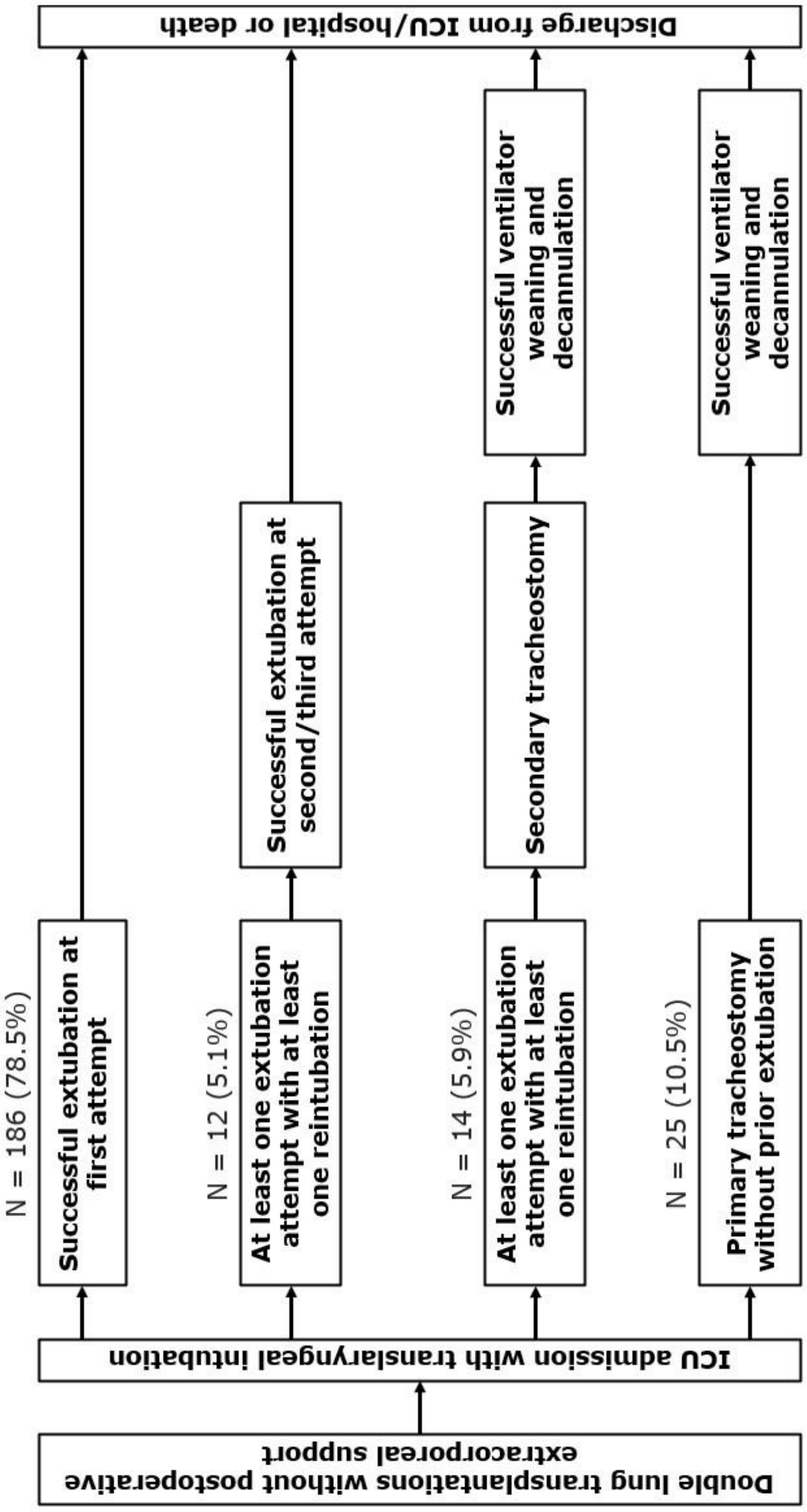

Table S1. Results of repeated measures ANOVA – within-subjects time effects

Variable & Group	Source	Sum of squares	DF	Mean square	F	*P* value
**LTC_{dyn}**						
All patients	Factor	361.506	3	120.502	8.60	**< 0.01**
	Residual	9925.994	708	14.020		
PMV > 72 hours	Factor	45.134	3	15.045	1.33	0.266
	Residual	2756.886	243	11.345		
Non-PMV	Factor	473.639	4	157.880	10.40	**< 0.01**
	Residual	7011.861	462	15.177		
**Mechanical power**						
All patients	Factor	336.513	3	112.171	11.61	**< 0.01**
	Residual	6837.594	708	9.658		
PMV > 72 hours	Factor	342.109	3	114.036	12.30	**< 0.01**
	Residual	2252.301	243	9.269		
Non-PMV	Factor	103.072	4	34.357	3.55	**0.015**
	Residual	4476.625	462	9.690		
**PBW-MP**						
All patients	Factor	0.0867	3	0.0289	12.40	**< 0.01**
	Residual	1.649	708	0.00233		
PMV > 72 hours	Factor	0.0922	3	0.0307	12.10	**< 0.01**
	Residual	0.617	243	0.00254		
Non-PMV	Factor	0.0246	4	0.00821	3.79	**0.011**
	Residual	1.002	462	0.00217		
**LTC_{dyn}-MP**						
All patients	Factor	54635405.072	3	18211801.691	9.30	**< 0.01**
	Residual	1386651674.000	708	1958547.563		
PMV > 72 hours	Factor	49828856.561	3	16609618.854	9.40	**< 0.01**
	Residual	429261210.000	243	1766507.037		
Non-PMV	Factor	31426988.219	4	10475662.740	5.20	**< 0.01**
	Residual	930770025.000	462	2014653.733		

Legend

Abbreviations: LTC_{dyn}, dynamic lung-thorax compliance; PMV, prolonged mechanical ventilation > 72 hours; PBW-MP, mechanical power normalized to the predicted body weight; LTC_{dyn}-MP, mechanical power normalized to dynamic lung-thorax compliance.

Table S2. Results of repeated measures ANOVA – between-group differences

Variable & Group	Source	Sum of squares	DF	Mean square	F	*P* value
**LTC_{dyn}**						
PMV > 72 hours	Factor	6785.245	1	6785.245	35.13	**< 0.01**
Non-PMV	Residual	45392.887	235	193.161		
**Mechanical power**						
PMV > 72 hours	Factor	2214.718	1	2214.718	27.29	**< 0.01**
Non-PMV	Residual	19074.044	235	81.166		
**PBW-MP**						
PMV > 72 hours	Factor	1.133	1	1.133	64.42	**< 0.01**
Non-PMV	Residual	4.133	235	0.0176		
**LTC_{dyn}-MP**						
PMV > 72 hours	Factor	1170405712.000	1	1170405712.000	66.64	**< 0.01**
Non-PMV	Residual	4127485379.272	235	17563767.571		

Legend

Abbreviations: LTC_{dyn}, dynamic lung-thorax compliance; PMV, prolonged mechanical ventilation > 72 hours; PBW-MP, mechanical power normalized to the predicted body weight; LTC_{dyn}-MP, mechanical power normalized to dynamic lung-thorax compliance.

Table S3. Correlations between ventilatory indexes and post-transplant invasive ventilation duration

Ventilatory indexes	Spearman`s correlation coefficient (ρ)	*P* value
P/F ratio	- 0.261 (- 0.376 – - 0.138)	**< 0.01**
Ventilatory ratio	0.188 (0.062 – 0.308)	**< 0.01**
LTC_{dyn}	- 0.360 (- 0.466 – - 0.244)	**< 0.01**
Mechanical power	0.252 (0.129 – 0.368)	**< 0.01**
PBW-MP	0.386 (0.272 – 0.489)	**< 0.01**
LTC_{dyn}-MP	0.452 (0.345 – 0.548)	**< 0.01**

Legend

Spearman`s correlation coefficient (ρ) of rank correlation (with 95% confidence intervals).

Abbreviations: P/F ratio, the ratio of partial pressure of oxygen to fractional inspired oxygen; LTC_{dyn}, dynamic lung-thorax compliance; PBW-MP, mechanical power normalized to the predicted body weight; LTC_{dyn}-MP, mechanical power normalized to dynamic lung-thorax compliance.

Table S4. Area under the ROC curve of ventilatory indexes regarding the prediction of post-transplant prolonged mechanical ventilation (> 72 hours)

Ventilatory indexes	Prolonged mechanical ventilation (> 72 hours)	*P* value
P/F ratio	0.69 (0.63 – 0.75)	**< 0.01**
Ventilatory ratio	0.65 (0.59 – 0.71)	**< 0.01**
LTC_{dyn}	0.72 (0.66 – 0.79)	**< 0.01**
Mechanical power	0.66 (0.60 – 0.72)	**< 0.01**
PBW-MP	0.76 (0.70 – 0.81)	**< 0.01**
LTC_{dyn}-MP	0.78 (0.72 – 0.83)	**< 0.01**

Legend

The accuracy of each index in predicting prolonged mechanical ventilation (> 72 hours following lung transplantation) presented as the area under the ROC curve with 95% confidence intervals.

Abbreviations: P/F ratio, the ratio of partial pressure of oxygen to fractional inspired oxygen; LTC_{dyn}, dynamic lung-thorax compliance; PBW-MP, mechanical power normalized to the predicted body weight; LTC_{dyn}-MP, mechanical power normalized to dynamic lung-thorax compliance.

Figure S2. Comparison of ROC curves for mechanical power and power density predicting post-transplant prolonged mechanical ventilation (> 72 hours)

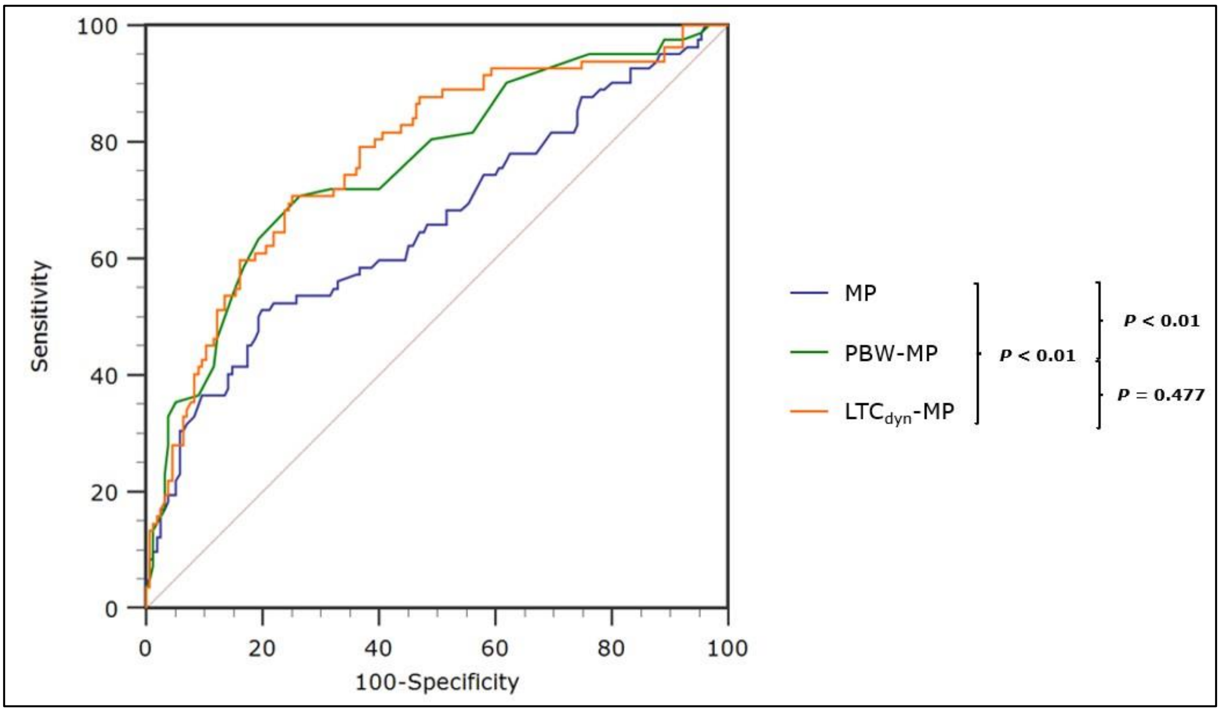

Legend

Comparison of the area under the receiver operating characteristic (ROC) curve (with 95% confidence intervals) for MP (0.66 [0.60–0.72]), PBW-MP (0.76 [0.70–0.81]), and LTC_{dyn}-MP (0.78 [0.72–0.83]).

Abbreviations: MP, mechanical power; PBW-MP, mechanical power normalized to the predicted body weight; LTC_{dyn}-MP, mechanical power normalized to dynamic lung-thorax compliance.

Table S5. Sensitivity analysis: Area under the ROC curve of ventilatory indexes regarding the prediction of post-transplant invasive mechanical ventilation > 96 hours

Ventilatory indexes	Invasive mechanical ventilation > 96 hours	*P* value
P/F ratio	0.71 (0.65 – 0.77)	**< 0.01**
Ventilatory ratio	0.68 (0.61 – 0.74)	**< 0.01**
LTC_{dyn}	0.70 (0.63 – 0.75)	**< 0.01**
Mechanical power	0.68 (0.61 – 0.74)	**< 0.01**
PBW-MP	0.76 (0.70 – 0.82)	**< 0.01**
LTC_{dyn}-MP	0.78 (0.72 – 0.83)	**< 0.01**

Legend

The accuracy of each index in predicting invasive mechanical ventilation > 96 hours following lung transplantation presented as the area under the ROC curve with 95% confidence intervals.

Abbreviations: P/F ratio, the ratio of partial pressure of oxygen to fractional inspired oxygen; LTC_{dyn}, dynamic lung-thorax compliance; PBW-MP, mechanical power normalized to the predicted body weight; LTC_{dyn}-MP, mechanical power normalized to dynamic lung-thorax compliance.

Table S6. Sensitivity analysis: Area under the ROC curve of ventilatory indexes regarding the prediction of post-transplant invasive mechanical ventilation > 7 days

Ventilatory indexes	Invasive mechanical ventilation > 7 days	*P* value
P/F ratio	0.74 (0.68 – 0.79)	**< 0.01**
Ventilatory ratio	0.73 (0.69 – 0.78)	**< 0.01**
LTC_{dyn}	0.69 (0.63 – 0.75)	**< 0.01**
Mechanical power	0.75 (0.69 – 0.80)	**< 0.01**
PBW-MP	0.80 (0.74 – 0.85)	**< 0.01**
LTC_{dyn}-MP	0.82 (0.77 – 0.87)	**< 0.01**

Legend

The accuracy of each index in predicting invasive mechanical ventilation > 7 days following lung transplantation presented as the area under the ROC curve with 95% confidence intervals.

Abbreviations: P/F ratio, the ratio of partial pressure of oxygen to fractional inspired oxygen; LTC_{dyn}, dynamic lung-thorax compliance; PBW-MP, mechanical power normalized to the predicted body weight; LTC_{dyn}-MP, mechanical power normalized to dynamic lung-thorax compliance.

Table S7. Sensitivity analysis: Correlations between ventilatory indexes and post-transplant invasive ventilation duration in patients successfully extubated at the first attempt (N = 186)

Ventilatory indexes	Spearman`s correlation coefficient (*ρ*)	*P* value
P/F ratio	- 0.095 (- 0.235 – 0.050)	0.199
Ventilatory ratio	0.029 (- 0.115 – 0.172)	0.693
LTC_{dyn}	- 0.295 (- 0.421 – - 0.157)	**< 0.01**
Mechanical power	0.119 (- 0.025 – 0.259)	0.105
PBW-MP	0.249 (0.109 – 0.379)	**< 0.01**
LTC_{dyn}-MP	0.313 (0.177 – 0.437)	**< 0.01**

Legend

Spearman`s correlation coefficient (*ρ*) of rank correlation (with 95% confidence intervals).

Abbreviations: P/F ratio, the ratio of partial pressure of oxygen to fractional inspired oxygen; LTC_{dyn}, dynamic lung-thorax compliance; PBW-MP, mechanical power normalized to the predicted body weight; LTC_{dyn}-MP, mechanical power normalized to dynamic lung-thorax compliance.

Table S8. Sensitivity analysis: Area under the ROC curve of ventilatory indexes regarding the prediction of post-transplant prolonged mechanical ventilation (> 72 hours) in patients successfully extubated at the first attempt (N = 186)

Ventilatory indexes	Prolonged mechanical ventilation (> 72 hours)	*P* value
P/F ratio	0.66 (0.58 – 0.72)	**< 0.01**
Ventilatory ratio	0.63 (0.56 – 0.70)	**< 0.01**
LTC_{dyn}	0.73 (0.66 – 0.79)	**< 0.01**
Mechanical power	0.64 (0.56 – 0.71)	**0.017**
PBW-MP	0.77 (0.70 – 0.83)	**< 0.01**
LTC_{dyn}-MP	0.76 (0.69 – 0.82)	**< 0.01**

Legend

The accuracy of each index in predicting prolonged mechanical ventilation (> 72 hours following lung transplantation) presented as the area under the ROC curve with 95% confidence intervals.

Abbreviations: P/F ratio, the ratio of partial pressure of oxygen to fractional inspired oxygen; LTC_{dyn}, dynamic lung-thorax compliance; PBW-MP, mechanical power normalized to the predicted body weight; LTC_{dyn}-MP, mechanical power normalized to dynamic lung-thorax compliance.

Table S9. Comparison of lung transplant recipients with different levels of respiratory support

Clinical characteristics	**1. Extubations without NIRS** (n = 117)	**2. Extubations with NIRS** (n = 95)	**3. Primary tracheostomy** (n = 25)
Age (years)	56 (50–61)	55 (41–60)	55 (51–59)
Height (m)	1.72 (1.67–1.78)	1.70 (1.65–1.76)	1.68 (1.60–1.77)
Predicted body weight (kg)	67 (60–73)	64 (57–71)	61 (52–72)
Body mass index (kg/m^2)	23.3 (19.8–26.1)[b]	22.2 (19.3–25.7)[c]	26.6 (20.1–29.1)
**Ventilatory variables**			
Respiratory rate (1/min)	17 (15–20)[b]	17 (15–20)[c]	20 (19–24)
Tidal volume (mL)	407 (367–452)	391 (346–438)	387 (333–436)
Tidal volume/recipient PBW (mL/kg)	6.2 (5.6–6.6)	6.0 (5.6–6.8)	6.1 (5.8–6.9)
Tidal volume/donor PBW (mL/kg)	5.8 (5.3–6.4)	5.7 (5.1–6.5)	5.6 (5.2–6.7)
Minute ventilation (L/min)	6.8 (5.7–8.1)[b]	6.7 (5.8–8.1)[c]	8.1 (6.5–10.2)
PEEP (cmH_2O)	9 (8–10)[b]	10 (8–10)[c]	11 (10–14)
P_{peak} (cmH_2O)	22 (21–24)[a/b]	23 (21–25)[c]	27 (25–28)
Dynamic driving pressure (cmH_2O)	13 (12–14)[a/b]	14 (12–15)[c]	16 (14–17)
**Ventilatory indexes**			
P/F ratio (mmHg)	301 (252–355)[a/b]	272 (226–322)[c]	192 (133–241)
Ventilatory ratio	1.17 (1.04–1.29)[b]	1.22 (1.05–1.38)[c]	1.56 (1.31–1.78)
LTC_{dyn} (mL/cmH_2O)	32 (26–38)[b]	30 (25–36)	26 (22–34)
Mechanical power (Joule/min)	14.7 (12.4–17.8)[b]	15.3 (12.5–19.1)[c]	20.6 (18.0–27.1)
PBW-MP (Joule/min/kg)	0.23 (0.19–0.26)[b]	0.24 (0.20–0.29)[c]	0.35 (0.29–0.39)
LTC_{dyn}-MP (cmH_2O^2/min)	4867 (3790–6222)[a/b]	5194 (4035–7543)[c]	7975 (6530–11094)
**Primary outcome**			
Duration of invasive ventilation (hours)	35.3 (22.0–52.7)[a/b]	53.0 (28.5–109)[c]	848 (594–1132)
**Secondary outcomes**			
Prolonged mechanical ventilation (> 72 h)	22 (18.8)[a/b]	35 (36.8)[c]	25 (100.0)
Admission to weaning readiness (hours)*	15.2 (9.4–23.3)[b]	17.2 (11.7–26.1)[c]	45.9 (31.6–65.6)
Reintubation	3 (2.6)[a]	21 (22.1)	–
Time to extubation (hours)	35.3 (22.1–52.0)[a]	44.7 (25.0–78.4)	–
Tracheostomy	2 (1.7)[a]	12 (12.6)	–
ICU length of stay (days)	5 (4–8)[a/b]	9 (6–19)[c]	42 (29–60)

Legend

A Chi-square test was used when comparing categorical variables, and differences in continuous variables were analyzed through Mann-Whitney *U*-test.

*: The time between ICU admission (while patients were on pressure-controlled ventilation) and switch to pressure support ventilation, indicating "*weaning readiness*".

a: Significant differences between Group 1 and 2 ($P < 0.05$)
b: Significant differences between Group 1 and 3 ($P < 0.05$)
c: Significant differences between Group 2 and 3 ($P < 0.05$)

Abbreviations: NIRS, non-invasive respiratory support (NIV and/or NHFC); COPD, chronic obstructive pulmonary disease; PBW, predicted body weight; P/F ratio, the quotient of partial pressure of oxygen to the fractional inspired oxygen; LTC_{dyn}, dynamic lung-thorax compliance; PBW-MP, mechanical power normalized to the predicted body weight; LTC_{dyn}-MP, mechanical power normalized to the dynamic lung-thorax compliance.

Table S10. Variables independently associated with prolonged mechanical ventilation (PMV) following double lung transplantation – Results of univariable and multivariable binary logistic regression analysis

Variables	Univariable analysis		Multivariable analysis	
	OR (95%CI)	*P value*	OR (95%CI)	*P value*
**Recipient characteristics**				
Age (years)	0.99 (0.97–1.01)	0.399	–	n.s.
Female gender	2.96 (1.70–5.16)	**< 0.01**	2.45 (1.29–4.66)	**< 0.01**
Body mass index (kg/m^2)	1.04 (0.97–1.10)	0.273	–	n.s.
**Recipient pulmonary hemodynamics**				
Mean pulmonary artery pressure (mmHg)	1.04 (1.01–1.07)	**< 0.01**	1.05 (1.02–1.09)	**< 0.01**
**Reason for lung transplantation**				
Interstitial lung diseases	1.16 (0.68–1.98)	0.593	–	n.s.
Pulmonary arterial hypertension	7.90 (0.87–71.9)	0.067	–	n.s.
**Transplant characteristics**				
Lung allocation score (points)	1.02 (0.99–1.04)	0.264	–	n.s.
pTLC ratio	1.87 (0.23–15.5)	0.561	–	n.s.
Undersized lungs (pTLC ratio < 0.9)	0.41 (0.11–1.50)	0.180	–	n.s.
Ischemic time left graft (hours)	1.00 (1.00–1.00)	0.274		
Ischemic time right graft (hours)	1.00 (1.00–1.00)	0.870	–	n.s.
Intraoperative ECMO	2.65 (1.50–4.68)	**< 0.01**	–	n.s.
RBC transfusion	2.24 (1.11–4.54)	**0.025**	–	n.s.
**Donor parameters**				
Age (years)	1.01 (1.00–1.03)	0.135	–	n.s.
Smoking history	1.28 (0.72–2.28)	0.406	–	n.s.
Antibiotics	1.39 (0.72–2.67)	0.328	–	n.s.
RBC transfusion	1.69 (0.90–3.18)	0.100	–	n.s.
Duration of invasive ventilation (days)	1.06 (1.00–1.13)	0.051	–	n.s.
P/F ratio at the time of organ offer (mmHg)	1.00 (1.00–1.00)	0.919	–	n.s.
P_aCO_2 at the time of organ offer (mmHg)	1.01 (0.97–1.05)	0.660	–	n.s.
**Ventilatory variables and mechanical power**				
P/F ratio * 10^{-2} (mmHg)	0.38 (0.26–0.56)	**< 0.01**	0.57 (0.36–0.88)	**0.012**
Mechanical power (Joule/min)	1.14 (1.08–1.21)	**< 0.01**	–	n.s.
LTC_{dyn}-MP * 10^{-3} (cmH_2O^2/min)	1.64 (1.40–1.92)	**< 0.01**	1.54 (1.30–1.83)	**< 0.01**

Legend

Recipient, transplant, and donor characteristics deemed clinically relevant a priori and those with *P* values less than 0.2 were used as input variables in the multivariable binary regression analysis.

Abbreviations: OR, odds ratio; 95%CI, 95% confidence interval; n.s., not significant; pTLC ratio, predicted total lung capacity ratio; ECMO, extracorporeal membrane oxygenation; RBC, packed red blood cell transfusions; P/F ratio, the quotient of partial pressure of oxygen to the fractional inspired oxygen; LTC_{dyn}-MP, mechanical power normalized to dynamic lung-thorax compliance.

Table S11. Multivariable binary logistic regression model

**Logit(p)**	$L = -3.411 + (0.896 * \text{female gender}) + (0.049 * \text{MPAP}) + (-0.567 * \text{P/F ratio} * 10^{-2}) + (0.432 * \text{LTC}_{dyn}\text{-MP} * 10^{-3})$			
**OR (95%CI); *P* value**	**Female gender**	2.45 (1.29–4.66)		< 0.01
	**MPAP**	1.05 (1.02–1.09)		< 0.01
	**P/F ratio * 10^{-2}**	0.57 (0.36–0.88)		0.012
	**LTC_{dyn}-MP * 10^{-3}**	1.54 (1.30–1.83)		< 0.01
**Hosmer & Lemeshow**	0.566			
**Nagelkerke R^2**	0.385			
**AUROC (95%CI)**	0.82 (0.77 – 0.87)			
**2 x 2 table**	**TP**<br>44		**FP**<br>17	
	**FN**<br>38		**TN**<br>138	
**Metrics from 2 x 2 table (95%CI)**	**Sensitivity**	54% (42–65)	**PLR**	4.89
	**Specificity**	89% (83–93)	**NLR**	0.52
	**PPV**	72% (61–81)	**DOR**	9.4
	**NPV**	78% (74–82)	**F_1 score**	0.62
	**Accuracy**	77% (71–82)	**MCC**	0.46

Legend

Variables independently associated with prolonged mechanical ventilation (PMV) following double lung transplantation after adjustment for: Recipient body mass index, intraoperative ECMO and red blood cell transfusion during transplant, interstitial lung diseases, and pulmonary artery hypertension as the primary reason for lung transplantation, pTLC ratio, and presence of an undersized allograft (pTLC < 0.90), a donor history of smoking or antibiotic treatment before transplant, donor P/F ratio and P_aCO_2 at the time of organ offer, duration of invasive ventilation in the donor before organ retrieval, Lung Allocation Score, and ischemic times of grafts.

Abbreviations: L, log-odds/Logit(p); MPAP, recipients` mean pulmonary artery pressure before transplant; P/F ratio, the quotient of partial pressure of oxygen to the fractional inspired oxygen; LTC_{dyn}-MP, mechanical power normalized to the dynamic lung-thorax compliance; OR, odds ratio; 95%CI, 95% confidence interval; AUROC, area under the receiver operating characteristic curve; TP, true positive; FP, false positive; FN, false negative; TN, true positive; PPV, positive predictive value; NPV, negative predictive value; PLR, positive likelihood ratio; NLR, negative likelihood ratio; DOR, diagnostic odds ratio; MCC, Matthews correlations coefficient[3]

Table S12. Comparison of patients with different primary graft dysfunction (PGD) grades at time-point T+72h after lung transplant

	Endotracheal intubation at T+72h								Extubated patients at T+72h	
**Clinical characteristics**	**PGD 3** (n = 18)	**PGD 2** (n = 28)	**PGD 1** (n = 11)	**PGD 0** (n = 22)	*P* value[a]	**PGD 1-2** (n = 39)	*P* value[b]	**PGD 1-3** (n = 57)	**All patients** (n = 158)	*P* value[c]
Age (years)	54 (51–59)	55 (47–58)	53 (32–56)	59 (54–61)	0.205[d]	54 (38–57)	0.399[d]	54 (44–58)	56 (47–61)	0.095[d]
Female gender	12 (66.7)	15 (53.6)	7 (63.6)	14 (63.6)	0.844[e]	22 (56.4)	0.467[e]	34 (59.6)	50 (31.6)	**< 0.01[e]**
Predicted body weight (kg)	55 (51–68)	62 (53–71)	52 (47–71)	60 (55–69)	0.157[d]	60 (52–71)	0.294[d]	57 (52–71)	68 (62–73)	**< 0.01[d]**
Body mass index (kg/m^2)	24.3 (20.1–28.1)	25.9 (19.2–27.5)	22.2 (17.8–25.2)	24.5 (19.8–27.1)	0.828[d]	23.7 (19.0–27.4)	0.440[d]	24.2 (19.5–27.5)	22.8 (19.6–25.7)	0.279[d]
**Recipient pulmonary hemodynamics**										
Mean pulmonary artery pressure (mmHg)	29 (25–42)	28 (22–32)	25 (23–28)	27 (21–36)	0.849[d]	27 (22–30)	0.340[d]	27 (23–34)	24 (20–30)	**0.012[d]**
Pulmonary vascular resistance (WU)	4.7 (2.2–6.1)	3.5 (2.4–5.7)	3.2 (2.3–4.4)	3.5 (2.7–6.0)	0.957[d]	3.4 (2.4–4.8)	0.307[d]	3.5 (2.3–5.5)	2.6 (1.9–3.7)	**< 0.01[d]**
**Transplant characteristics**										
pTLC ratio	1.07 (1.01–1.13)	1.08 (0.99–1.14)	1.03 (1.00–1.17)	1.05 (0.96–1.14)	0.775[d]	1.07 (0.99–1.14)	0.588[d]	1.07 (1.00–1.13)	1.06 (0.98–1.12)	0.623[d]
Undersized lungs (pTLC ratio < 0.9)	0 (0.0)	1 (3.6)	0 (0.0)	2 (9.1)	0.195[e]	1 (2.6)	0.497[e]	1 (1.8)	13 (8.2)	0.090[e]
Intraoperative ECMO	14 (77.8)	19 (67.9)	9 (81.8)	15 (68.2)	0.504[e]	28 (71.8)	0.637[e]	42 (73.7)	75 (47.5)	**< 0.01[e]**
RBC transfusion	15 (83.3)	23 (82.1)	10 (90.9)	19 (86.4)	0.792[e]	33 (84.6)	0.903[e]	48 (84.2)	115 (72.8)	0.085[e]
**Donor parameters**										
Age (years)	55 (50–59)	46 (38–64)	47 (25–60)	38 (22–57)	**0.031[d]**	46 (37–63)	**0.035[d]**	50 (41–62)	45 (31–58)	**0.030[d]**
Smoking history	8 (44.4)	11 (39.3)	1 (9.1)	6 (27.3)	0.263[e]	12 (30.8)	0.319[e]	20 (35.1)	44 (27.8)	0.307[e]
RBC transfusion	4 (22.2)	5 (17.9)	6 (54.5)	7 (31.8)	0.504[e]	11 (28.2)	0.637[e]	15 (26.3)	30 (19.0)	0.245[e]
Duration of invasive ventilation (days)	4 (3–6)	4 (3–7)	4 (2–6)	3 (2–7)	0.378[d]	4 (3–6)	0.721[d]	4 (3–6)	3 (2–5)	0.077[d]
P/F ratio at the time of organ offer (mmHg)	423 (378–502)	458 (396–511)	423 (407–448)	409 (368–483)	0.968[d]	442 (400–505)	0.250[d]	432 (387–503)	429 (373–482)	0.478[d]
**Ventilatory variables**										
Respiratory rate (1/min)	20 (19–22)	18 (17–20)	18 (16–20)	19 (18–22)	0.731[d]	18 (17–20)	0.118[d]	19 (17–20)	17 (15–19)	**< 0.01[d]**
Tidal volume (mL)	391 (320–434)	387 (337–436)	372 (324–401)	376 (347–402)	0.505[d]	374 (334–433)	0.706[d]	386 (333–433)	409 (368–455)	**0.015[d]**
Tidal volume/recipient (mL/kg)	6.3 (5.7–7.4)	6.4 (5.6–6.6)	6.4 (5.7–7.2)	6.1 (6.0–6.7)	0.619[d]	6.4 (5.6–6.7)	0.354[d]	6.4 (5.7–7.0)	6.1 (5.6–6.7)	0.168[d]
Tidal volume/donor PBW (mL/kg)	5.9 (5.2–6.7)	5.9 (5.1–6.5)	5.4 (5.2–7.4)	5.7 (5.1–6.8)	0.532[d]	5.8 (5.1–6.6)	0.440[d]	5.8 (5.2–6.6)	5.8 (5.2–6.4)	0.657[d]
Minute ventilation (L/min)	7.8 (6.1–9.0)	7.3 (6.1–8.1)	6.2 (5.4–7.7)	7.5 (5.8–8.4)	0.683[d]	7.2 (5.7–7.9)	0.233[d]	7.3 (5.8–8.5)	6.8 (5.8–8.1)	0.388[d]
PEEP (cmH_2O)	10 (8–12)	10 (9–12)	10 (9–10)	12 (10–14)	0.114[d]	10 (9–12)	0.570[d]	10 (9–12)	9 (8–10)	**< 0.01[d]**
P_{peak} (cmH_2O)	25 (23–28)	26 (24–28)	26 (22–27)	27 (24–28)	0.338[d]	26 (23–27)	0.924[d]	25 (23–28)	22 (20–24)	**< 0.01[d]**
Dynamic driving pressure (cmH_2O)	16 (13–17)	15 (13–16)	14 (13–17)	15 (14–16)	0.380[d]	15 (13–16)	0.446[d]	15 (13–17)	13 (11–14)	**< 0.01[d]**
**Ventilatory indexes**										
P/F ratio (mmHg)	190 (136–245)	248 (220–315)	300 (246–351)	228 (144–293)	0.253[d]	266 (224–322)	**< 0.01[d]**	239 (192–315)	296 (253–344)	**< 0.01[d]**
Ventilatory ratio	1.33 (1.20–1.64)	1.30 (1.16–1.42)	1.24 (0.98–1.45)	1.37 (1.21–1.68)	0.978[d]	1.29 (1.13–1.42)	0.099[d]	1.30 (1.17–1.47)	1.18 (1.03–1.31)	**< 0.01[d]**
LTC_{dyn} (mL/cmH_2O)	28 (20–34)	27 (23–31)	24 (21–31)	26 (22–28)	0.653[d]	27 (22–31)	0.790[d]	27 (22–32)	32 (28–38)	**< 0.01[d]**
Mechanical power (Joule/min)	18.1 (14.0–21.2)	16.4 (14.1–21.1)	12.9 (11.2–19.7)	18.9 (15.1–22.9)	0.796[d]	16.3 (12.9–21.0)	0.420[d]	18.0 (13.6–21.1)	14.7 (12.1–17.3)	**< 0.01[d]**
PBW-MP (Joule/min/kg)	0.31 (0.26–0.37)	0.29 (0.23–0.34)	0.26 (0.23–0.30)	0.33 (0.27–0.38)	0.849[d]	0.28 (0.23–0.33)	0.068[d]	0.29 (0.23–0.35)	0.22 (0.19–0.26)	**< 0.01[d]**
LTC_{dyn}-MP (cmH_2O^2/min)	6910 (6142-9897)	6892 (5185-8136)	6341 (5260-7368)	7251 (5882-8299)	0.999[d]	6504 (5198-8051)	0.287[d]	6773 (5207-8217)	4417 (3769-5980)	**< 0.01[d]**
**Primary outcome**										
Duration of invasive ventilation (hours)	603 (108–867)	152 (87.2–601)	117 (93.4–236)	438 (131–848)	0.989[d]	138 (89.2–351)	**0.023[d]**	161 (95.9–644)	33.0 (20.8–45.8)	**< 0.01[d]**
**Secondary outcomes**										
Prolonged mechanical ventilation (> 72 h)	18 (100.0)	25 (89.3)	11 (100.0)	21 (95.5)	0.366[e]	36 (92.6)	0.231[e]	54 (94.7)	7 (4.4)	**< 0.01[e]**
Admission to weaning readiness (hours)*	36.4 (23.2–61.1)	23.8 (12.9–34.0)	39.5 (21.5–51.4)	32.7 (22.2–45.4)	0.443[d]	27.2 (16.6–43.5)	0.114[d]	28.9 (16.9–47.9)	14.3 (9.4–20.9)	**< 0.01[d]**
Extubation	9 (50.0)	23 (82.1)	10 (90.9)	13 (59.1)	0.570[e]	33 (84.6)	**< 0.01[e]**	42 (73.7)	157 (99.4)	**< 0.01[e]**
Time to extubation (hours)	106 (68.7–120)	100 (77.4–142)	93.8 (78.4–112)	129 (93.1–142)	0.292[d]	99.0 (78.3–130)	0.842[d]	99.7 (78.1–128)	32.7 (21.0–44.6)	**< 0.01[d]**
Reintubation	3 (33.3)	7 (30.4)	3 (33.3)	4 (30.8)	0.901[e]	10 (31.2)	0.907[e]	13 (31.7)	7 (4.5)	**< 0.01[e]**
within < 72 hours	2 (22.2)	5 (21.7)	2 (20.0)	3 (23.1)	0.963[e]	7 (21.2)	0.948[e]	9 (21.4)	2 (1.3)	**< 0.01[e]**
Tracheostomy	11 (61.1)	10 (35.7)	3 (27.3)	12 (54.5)	0.680[e]	13 (33.3)	0.050[e]	24 (42.1)	3 (1.9)	**< 0.01[e]**
ICU length of stay (days)	40 (11–52)	20 (10–43)	17 (9–21)	31 (13–63)	0.624[d]	19 (9–31)	0.091[d]	21 (9–43)	5 (4–8)	**< 0.01[d]**

Legend

a: P value for differences between *PGD grade 3* and *PGD grade 0* at T+72h hours after lung transplant.

b: P value for differences between *PGD grade 3* and *PGD grades 1-2* at T+72h hours after lung transplant.

c: *P* value for differences between *PGD grades 1-3* (mechanically ventilated) and *extubated patients* at T+72h hours after lung transplant.

d: Mann-Whitney *U*-test
e: Chi-squared test

*: The time between ICU admission (pressure-controlled ventilation) and switch to pressure support ventilation, indicating "*weaning readiness*".

Abbreviations: PGD, primary graft dysfunction; pTLC ratio, donor/recipient predicted total lung capacity ratio; ECMO, extracorporeal membrane oxygenation; RBC, packed red blood cell transfusions; P/F ratio, the quotient of partial pressure of oxygen to the fractional inspired oxygen; PBW, predicted body weight; PEEP, positive end-expiratory pressure; P_{peak}, peak inspiratory airway pressure; LTC_{dyn}, dynamic lung-thorax compliance; PBW-MP, mechanical power normalized to the predicted body weight; LTC_{dyn}-MP, mechanical power normalized to the dynamic lung-thorax compliance.

Table S13. Comparison of female and male lung transplant recipients

Clinical characteristics	All patients (n = 237)	Females (n = 98)	Males (n = 139)	*P* value[a]
Age (years)	56 (47–60)	56 (40–60)	56 (49–60)	0.249[b]
Height (m)	1.71 (1.65–1.78)	1.64 (1.59–1.68)	1.76 (1.72–1.82)	**< 0.01[b]**
Predicted body weight (kg)	66 (57–73)	56 (52–60)	71 (67–77)	**< 0.01[b]**
Body mass index (kg/m^2)	23.0 (19.6–26.6)	21.7 (18.7–24.8)	23.9 (20.6–27.3)	**< 0.01[b]**
**Ventilatory variables**				
Respiratory rate (1/min)	18 (15–20)	18 (15–20)	17 (15–20)	0.780[b]
Tidal volume (mL)	402 (349–442)	355 (320–394)	428 (392–476)	**< 0.01[b]**
Tidal volume/recipient PBW (mL/kg)	6.2 (5.6–6.8)	6.4 (5.9–7.0)	6.0 (5.5–6.5)	**< 0.01[b]**
Tidal volume/donor PBW (mL/kg)	5.9 (5.2–6.5)	5.9 (5.2–6.7)	5.7 (5.2–6.2)	0.118[b]
Minute ventilation (L/min)	6.9 (5.8–8.1)	6.0 (5.5–7.3)	7.6 (6.4–8.7)	**< 0.01[b]**
PEEP (cmH_2O)	10 (8–10)	10 (8–10)	10 (8–10)	0.653[b]
P_{peak} (cmH_2O)	23 (21–25)	24 (21–26)	23 (21–25)	**0.014[b]**
Dynamic driving pressure (cmH_2O)	13 (12–15)	14 (12–16)	13 (12–15)	**< 0.01[b]**
**Ventilatory indexes**				
P/F ratio (mmHg)	278 (225–338)	267 (209–329)	295 (232–341)	0.052[b]
Ventilatory ratio	1.21 (1.05–1.39)	1.24 (1.09–1.47)	1.20 (1.04–1.34)	**0.019[b]**
LTC_{dyn} (mL/cmH_2O)	31 (25–36)	26 (22–31)	33 (29–39)	**< 0.01[b]**
Mechanical power (Joule/min)	15.3 (12.6–19.1)	14.0 (11.8–17.1)	16.4 (13.6–20.5)	**< 0.01[b]**
PBW-MP (Joule/min/kg)	0.24 (0.20–0.29)	0.26 (0.21–0.32)	0.23 (0.19–0.28)	**< 0.01[b]**
LTC_{dyn}-MP (cmH_2O^2/min)	5328 (3999–7092)	5850 (4112–7870)	4947 (3973–6351)	**0.043[b]**
**Primary outcome**				
Duration of invasive ventilation (hours)	45.8 (24.8–109)	66.9 (25.9–175)	41.4 (23.4–67.0)	**0.010[b]**
**Secondary outcomes**				
Prolonged mechanical ventilation (> 72 h)	82 (34.6)	48 (49.0)	34 (24.5)	**< 0.01[c]**
Admission to weaning readiness (hours)*	17.4 (11.4–27.4)	17.5 (10.8–29.9)	17.3 (11.7–25.0)	0.632[b]
Extubation	212 (89.5)	84 (85.7)	128 (92.1)	0.117[c]
Time to extubation (hours)	39.9 (23.3–65.0)	43.1 (24.2–83.8)	37.9 (22.7–57.4)	0.184[b]
Non-invasive respiratory support[#]	95 (44.8)	42 (50.0)	53 (41.4)	0.220[c]
NHFC	59 (24.9)	29 (29.6)	30 (21.6)	0.161[c]
NIV	83 (35.0)	34 (34.7)	49 (35.2)	0.930[c]
Duration of NIRS (hours)	50.5 (28.5–81.9)	46.6 (25.2–88.7)	57.3 (34.1–79.2)	0.389[b]
Reintubation	24 (11.4)	15 (18.1)	9 (7.1)	**0.015[c]**
within < 72 hours	14 (6.6)	10 (11.9)	4 (3.1)	**0.013[c]**
Tracheostomy	39 (16.5)	23 (23.5)	16 (11.5)	**0.015[c]**
ICU length of stay (days)	7 (5–15)	9 (6–21)	6 (4–11)	**< 0.01[b]**

Legend

a: P value for differences between female and male lung transplant recipients

b: Mann-Whitney *U*-test

c: Chi-squared test

*: The time between ICU admission (while patients were on pressure-controlled ventilation) and switch to pressure support ventilation, indicating "*weaning readiness*".

[#]: NIV and/or NHFC use within 24 hours following (first) extubation for at least six hours daily.

Abbreviations: COPD, chronic obstructive pulmonary disease; PBW, predicted body weight; PEEP, positive end-expiratory pressure; P_{peak}, peak inspiratory airway pressure; P/F ratio, the quotient of partial pressure of oxygen to the fractional inspired oxygen; LTC_{dyn}, dynamic lung-thorax compliance; PBW-MP, mechanical power normalized to the predicted body weight; LTC_{dyn}-MP, mechanical power normalized to the dynamic lung-thorax compliance; NIRS, non-invasive respiratory support (NIV and/or NHFC); NHFC, nasal high flow cannula; NIV, non-invasive ventilation.

References

1. Sinha P, et al. Ventilatory ratio: a simple bedside measure of ventilation. *Br J Anaesth* 2009;102:692–697.
2. Sinha P, et al. Analysis of ventilatory ratio as a novel method to monitor ventilatory adequacy at the bedside. *Crit Care* 2013;17:R34.
3. Gattinoni L, et al. Ventilator-related causes of lung injury: the mechanical power. *Intensive Care Med* 2016;42:1567–1575.
4. Becher T, et al. Calculation of mechanical power for pressure-controlled ventilation. *Intensive Care Med* 2019;45:1321–1323.
5. Chiumello D, et al. Bedside calculation of mechanical power during volume- and pressure-controlled mechanical ventilation. *Crit Care* 2020;24:417.
6. Ghiani A, et al. Mechanical power normalized to lung-thorax compliance indicates weaning readiness in prolonged ventilated patients. *Sci Rep* 2022;12(1):6.
